# Pathogenic human variant that dislocates GATA2 zinc fingers disrupts hematopoietic gene expression and signaling networks

**DOI:** 10.1172/JCI162685

**Published:** 2023-04-03

**Authors:** Mabel Minji Jung, Siqi Shen, Giovanni A. Botten, Thomas Olender, Koichi R. Katsumura, Kirby D. Johnson, Alexandra A. Soukup, Peng Liu, Qingzhou Zhang, Zena D. Jensvold, Peter W. Lewis, Robert A. Beagrie, Jason K.K. Low, Lihua Yang, Joel P. Mackay, Lucy A. Godley, Marjorie Brand, Jian Xu, Sunduz Keles, Emery H. Bresnick

**Affiliations:** 1 Wisconsin Blood Cancer Research Institute, Department of Cell and Regenerative Biology, Carbone Cancer Center, and; 2 Department of Biostatistics and Biomedical Informatics, University of Wisconsin School of Medicine and Public Health, Madison, Wisconsin, USA.; 3 Children’s Medical Center Research Institute, Department of Pediatrics, University of Texas Southwestern Medical Center, Dallas, Texas, USA.; 4 Sprott Center for Stem Cell Research, Ottawa Hospital Research Institute–General Hospital, Ottawa, Ontario, Canada.; 5 Department of Biomolecular Chemistry, University of Wisconsin School of Medicine and Public Health, Madison, Wisconsin, USA.; 6 MRC Weatherall Institute of Molecular Medicine, Radcliffe Department of Medicine, University of Oxford, Oxford, United Kingdom.; 7 School of Life and Environmental Sciences, University of Sydney, Sydney, New South Wales, Australia.; 8 Section of Hematology/Oncology, The University of Chicago, Chicago, Illinois, USA.; 9 Department of Cell and Regenerative Biology, University of Wisconsin School of Medicine and Public Health, Madison, Wisconsin, USA.

**Keywords:** Genetics, Hematology, Genetic variation, Signal transduction, Transcription

## Abstract

Although certain human genetic variants are conspicuously loss of function, decoding the impact of many variants is challenging. Previously, we described a patient with leukemia predisposition syndrome (GATA2 deficiency) with a germline *GATA2* variant that inserts 9 amino acids between the 2 zinc fingers (9aa-Ins). Here, we conducted mechanistic analyses using genomic technologies and a genetic rescue system with *Gata2* enhancer–mutant hematopoietic progenitor cells to compare how GATA2 and 9aa-Ins function genome-wide. Despite nuclear localization, 9aa-Ins was severely defective in occupying and remodeling chromatin and regulating transcription. Variation of the inter–zinc finger spacer length revealed that insertions were more deleterious to activation than repression. GATA2 deficiency generated a lineage-diverting gene expression program and a hematopoiesis-disrupting signaling network in progenitors with reduced granulocyte-macrophage colony-stimulating factor (GM-CSF) and elevated IL-6 signaling. As insufficient GM-CSF signaling caused pulmonary alveolar proteinosis and excessive IL-6 signaling promoted bone marrow failure and GATA2 deficiency patient phenotypes, these results provide insight into mechanisms underlying GATA2-linked pathologies.

## Introduction

In metal-binding proteins, the metal requirement to regulate protein structure/function links mechanisms governing metal homeostasis, proteome composition, and genome regulation. Hundreds of proteins harbor zinc “fingers” that coordinate zinc ions (1). These proteins include transcription factors, in which the finger mediates DNA and/or protein binding. Sequences within one or more fingers confer sequence-specific DNA binding (1). Artificial zinc finger proteins can be engineered with designer DNA binding specificities for experimental and therapeutic applications (2). Finger-altering genetic variation can change DNA binding specificity (3) and cause diseases (4–8). Sequences extrinsic to fingers can enhance DNA-binding affinity (9). Insertion of KTS residues between Wilms tumor 1 zinc fingers via alternative splicing alters DNA binding specificity (10). Unlike sophisticated zinc finger structure/function knowledge, the impact of spacer length and sequence and alterations thereof cannot be predicted.

GATA transcription factors (11) harbor N- and C-zinc fingers, based on proximity to N- and C-termini (12, 13). In GATA1, which promotes erythroid, megakaryocytic, and mast cell development, the C-finger binds a small subset of WGATAR motifs (14, 15) in chromatin (16, 17). The N-finger binds the 9–zinc finger coregulator Friend of GATA1 (FOG1) (18, 19). Although FOG1 fingers do not appear to bind DNA, four are implicated in binding GATA1 (20). GATA2 promotes hematopoietic stem cell emergence in the embryo (21, 22) and hematopoiesis in adult mice (23, 24). Heterozygous *GATA2* coding or enhancer germline mutations cause GATA2 deficiency syndrome involving immunodeficiency, myelodysplastic syndrome, and acute myeloid leukemia (25–29). Most mutations alter the GATA2 C-finger (30) and inhibit DNA binding (27, 31) and chromatin occupancy (32, 33). Although *GATA2* mutational analyses revealed loss-of-function phenotypes that may underlie pathogenesis (30), GATA2 variants can partially retain or have supraphysiological activity (33, 34). GATA2 dysregulation is also implicated in myeloproliferative neoplasms (35).

We described a human variant that alters the distance between GATA2 zinc fingers. A family harbored a germline in-frame 9–amino acid insertion (9aa-Ins) between the N- and C-fingers (36). The 8-year-old proband had GATA2 deficiency with warts, lymphedema, and cytopenias, and the variant was detected in the asymptomatic mother. 9aa-Ins was defective in activating three GATA2 target genes tested. As GATA factor target genes are regulated via distinct mechanisms with context-dependent coregulator requirements (37), it is unpredictable whether 9aa-Ins would be defective in activating and repressing all target genes, whether defects reflect an inability to occupy and/or remodel chromatin, and whether it has activities distinct from GATA2 (ectopic). It is unclear whether 9aa-Ins defects would be recapitulated with any spacing alteration, whether select changes are tolerable, and whether a threshold exists beyond which alterations are inhibitory.

We analyzed how GATA2 and 9aa-Ins regulate chromatin and transcription genome-wide in a rescue system with GATA2-deficient progenitors. 9aa-Ins was defective in occupying and remodeling chromatin and regulating most target genes. GATA2 repressed expression of *Ebf1*, encoding a B lineage–regulatory transcription factor (38) and EBF1 target genes important in B cell biology, indicating a lineage-diverting expression program in GATA2-deficient fetal progenitors. *Csf2rb*, encoding the common β chain of granulocyte-macrophage colony-stimulating factor (GM-CSF), interleukin-3 (IL-3), and IL-5 receptors (39–41), exemplified a GATA2-activated gene compromised by the insertion, and this defect diminished GM-CSF signaling. In humans, defective GM-CSF signaling impairs the capacity of lung macrophages to consume surfactant, causing pulmonary alveolar proteinosis (42, 43), a severe GATA2 deficiency syndrome phenotype (44, 45). Genes encoding IL-6 receptor subunits (*Il6ra* and *Il6st*) (46, 47) were upregulated in GATA2-deficient cells, instigating IL-6 signaling, and 9aa-Ins did not repress expression. Ectopic IL-6 signaling drives immune dysregulation and bone marrow failure (48–50). Our analysis of a disease variant unveiled principles of GATA factor function and pathogenic mechanisms and will enable clinical genetic variant curation.

## Results

### GATA2 pathogenic variant with dislocated zinc fingers is severely defective genome-wide, yet retains activity at select loci.

**Figure 1:**
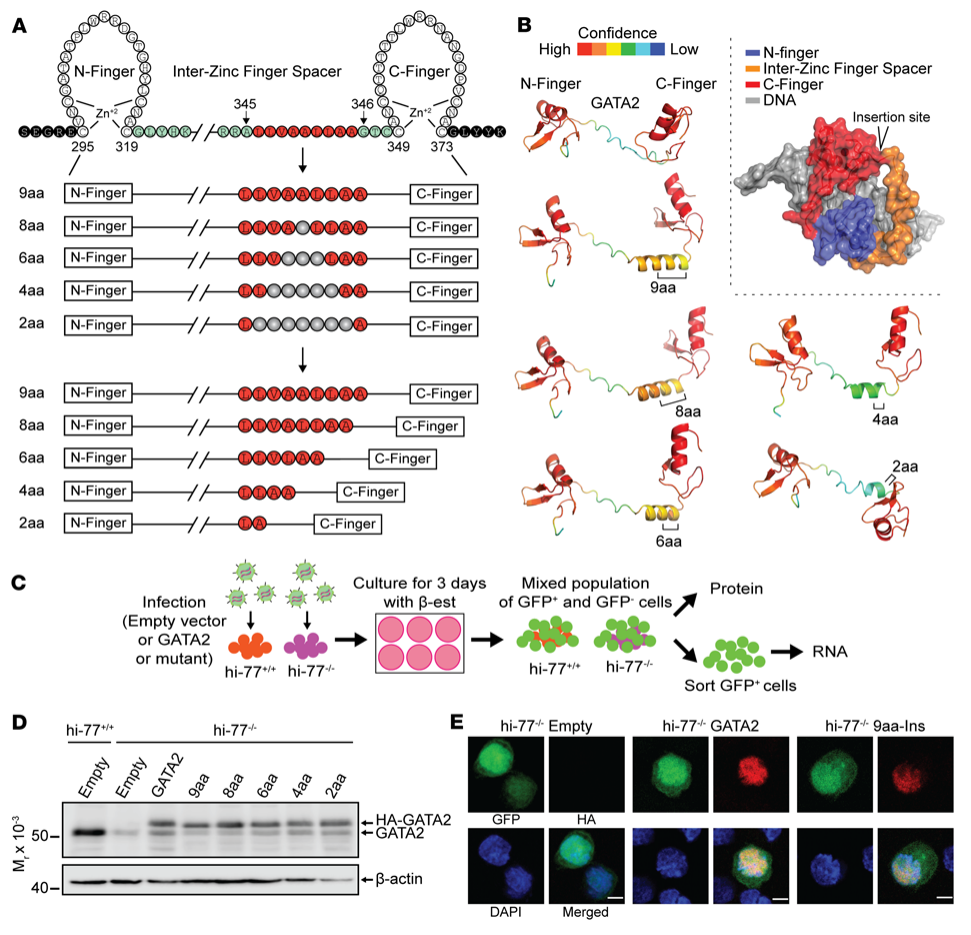
Artificial GATA2 transcription factors with variable inter–zinc finger spacers. (**A**) Mouse GATA2 9aa, 8aa, 6aa, 4aa, and 2aa insertion variants. The 9aa-Ins spacer variant models a human disease mutation (36). (**B**) AlphaFold prediction of structures. Model of GATA2 insertion site relative to the C-finger and N-finger. (**C**) Rescue assay with HoxB8-immortalized *Gata2* hi–77^+/+^ and mutant (hi–77^–/–^) cells. β-est, β-estradiol. (**D**) Western blot with anti-GATA2 antibody of hi–77 cells expressing endogenous GATA2 with or without HA-tagged GATA2 or variants (*n* = 9). (**E**) Immunofluorescence analysis of HA-GATA2 localization in hi–77 cells. Scale bars: 5 μm.

The GATA2 pathogenic 9-aa insertion (p.A345delinsALLVAALLAA) lies within the spacer between zinc fingers proximal to the DNA-binding C-finger (Figure 1A). The GATA2 zinc finger and spacer sequences are conserved (Supplemental Figure 1A; supplemental material available online with this article; https://doi.org/10.1172/JCI162685DS1), and spacer sequence and length are conserved among the mammalian GATA factors. To test whether the 9aa insertion is uniquely deleterious, we generated retroviruses to express variants with reductions (8, 6, 4, and 2 aa) from the middle of 9aa-Ins (X = deleted aa; LLVAXLLAA, LLVXXXLAA, LLXXXXXAA, LXXXXXXXA).

To assess whether the insertion alters the zinc finger domain conformation, we used the AlphaFold Protein Structure Database to predict structures of WT and 9aa-Ins variant domains (Figure 1B). The WT fingers display conformations similar to experimentally derived structures of other GATA zinc fingers (51–54). The spacer conformation is extended and predicted with low confidence, consistent with being disordered in solution and not discernible in crystal structures of GATA zinc finger proteins bound to DNA (51, 53). The 9aa insertion is predicted with intermediate confidence to form an α helix and induce 3 residues (RRA) from the WT spacer to adopt helicity (Figure 1B). This predicted helix was also observed with smaller insertion variants, with 8aa predicted to yield the longest helix and 2aa the smallest. Such a conformation might alter or generate GATA2-dependent macromolecular interactions (Figure 1B).

Given the invariant spacer, spacer length or sequence deviations might destabilize GATA2. We tested whether variants can be expressed in progenitors. We adapted our rescue assay with *Gata2*–77^–/–^ primary fetal liver progenitors (33, 36) to HoxB8-immortalized mutant progenitors (hi–77^–/–^) (55) (Figure 1C). WT and mutant hi–77^–/–^ cells were infected with control (empty) GFP-expressing retrovirus or retroviruses expressing GFP and HA-GATA2 or a variant. After culturing for 3 days, the population containing GFP^+^ cells was analyzed by Western blotting (Figure 1D and Supplemental Figure 1B). The mean infection efficiency quantified by flow cytometry was 27% GFP^+^ (Supplemental Figure 1C). Endogenous GATA2 mRNA and protein expression was approximately 75% lower in hi–77^–/–^ versus hi–77^+/+^ progenitors, consistent with –77 function to increase *Gata2* transcription in progenitors (55–57). As the HA tag reduced GATA2 mobility, anti-GATA2 antibody detected endogenous and exogenous GATA2. HA-tagged 9, 8, 6, 4, and 2aa variant levels resembled HA-GATA2 (Figure 1D) and were not destabilizing. Endogenous GATA2 levels in hi–77^+/+^ cells resembled HA-GATA2 or variant levels in hi–77^–/–^ (Supplemental Figure 1D). Using anti-HA to detect GATA2 or 9aa-Ins after expression in hi–77^–/–^ cells and GFP to identify infected hi–77^–/–^ cells, anti-HA staining in confocal microscopy was nuclear for GATA2 and 9aa-Ins (Figure 1E), with no immunoreactivity detected in control cells.

**Figure 2:**
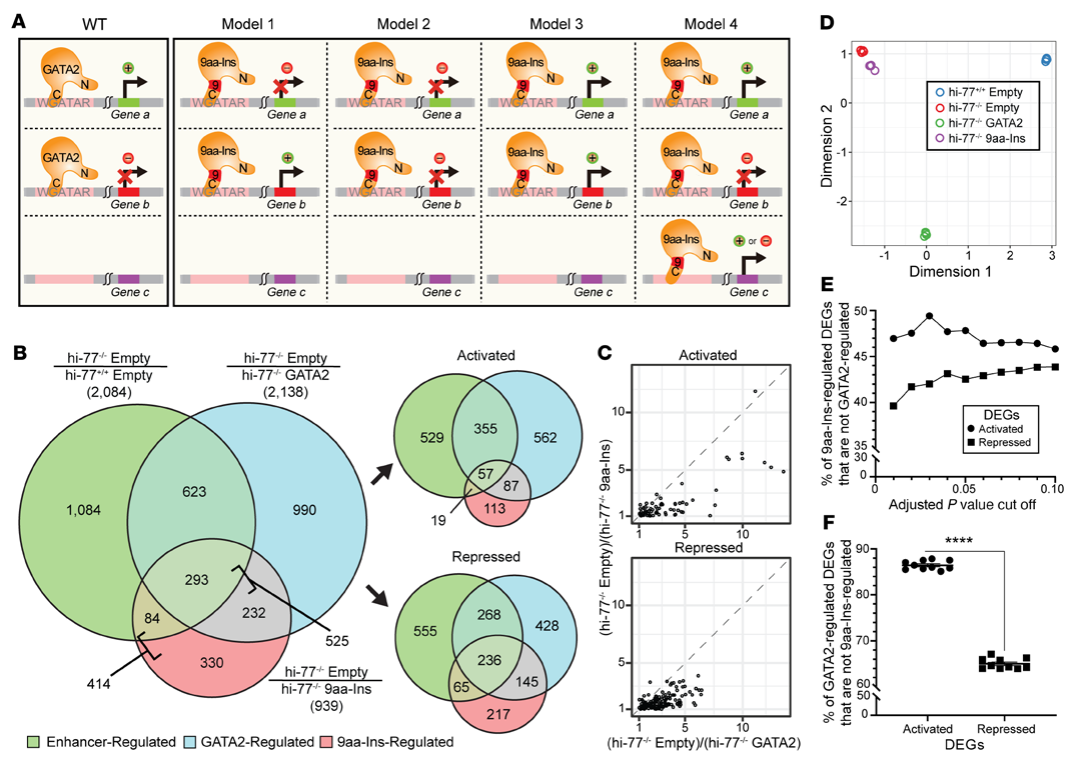
GATA2 9aa-Ins disease variant is severely defective, but not entirely inactive, in genome regulation. (**A**) Model 1, 9aa-Ins fails to regulate all GATA2-regulated genes; model 2, 9aa-Ins fails to repress GATA2-regulated genes; model 3, 9aa-Ins fails to activate GATA2-regulated genes; model 4, 9aa-Ins ectopically regulates genes that are not regulated by GATA2. (**B**) Overlap of DEGs that are –77-, GATA2-, and 9aa-Ins–regulated. RNA-Seq (4 biological replicates) of hi–77^+/+^ with control vector (hi–77^+/+^ empty), hi–77^–/–^ with control vector (hi–77^–/–^ empty), hi–77^–/–^ with GATA2 (hi–77^–/–^ GATA2), and hi–77^–/–^ with 9aa-Ins (hi–77^–/–^ 9aa-Ins). DEGs in hi–77^+/+^ empty, hi–77^–/–^ GATA2, and hi–77^–/–^ 9aa-Ins were defined as |log_2_(fold change)| ≥ 1 and adjusted *P* value < 0.05 relative to hi–77^–/–^ empty. Each circle represents DEGs in the 3 categories: green circle, enhancer-regulated, (hi–77^–/–^ empty)/(hi–77^+/+^ empty); blue circle, GATA2-regulated, (hi–77^–/–^ empty)/(hi–77^–/–^ GATA2); pink circle, 9aa-Ins–regulated, (hi–77^–/–^ empty)/(hi–77^–/–^ 9aa-Ins). DEGs were parsed into activated or repressed. DEG numbers are shown in parentheses. (**C**) Correlation plots depicting retention of 9aa-Ins–mediated activation and repression relative to GATA2. Comparison was calculated using |log_2_(fold change)| ≥ 1 of (hi–77^–/–^ empty)/(hi–77^–/–^ GATA2) and (hi–77^–/–^ empty)/(hi–77^–/–^ 9aa-Ins). Genes were required to have TPM ≥1 in all replicates in hi–77^–/–^ GATA2 for activation and hi–77^–/–^ empty for repression. (**D**) PCA quantifying multidimensional scaling distances between transcriptomes (4 biological replicates). (**E**) Percentage of 9aa-Ins–regulated genes that are not GATA2-regulated analyzed by subtraction of overlap of (hi–77^–/–^ empty)/(hi–77^–/–^ GATA2) from (hi–77^–/–^ empty)/(hi–77^–/–^ 9aa-Ins). *P* cutoffs ranged from 0.01 to 0.1. (**F**) Percentage of GATA2-regulated genes that are not 9aa-Ins–regulated analyzed by subtraction of (hi–77^–/–^ empty)/(hi–77^–/–^ 9aa-Ins) from (hi–77^–/–^ empty)/(hi–77^–/–^ GATA2). The same 10 *P* cutoffs as in **E** were used. The percentages of DEGs for each cutoff were parsed into activated or repressed. Statistical calculations used Mann-Whitney *U* test; ****P < 0.0001.

Previously, we demonstrated that 9aa-Ins is defective in activating 3 GATA2 target genes and promoting myeloerythroid progenitor activity when expressed in –77^–/–^ progenitors (36). As context-dependent GATA factor mechanisms preclude predictions at a given locus (11, 37), and genetic variation can create ectopic functions (58), the relationship between defective 9aa-Ins activity and genome-wide function was unclear. We tested whether 9aa-Ins was defective in activation and/or repression and/or acquires ectopic activity. With the rescue assay, we expressed GATA2 or 9aa-Ins, isolated GFP^+^ cells, and used RNA-Seq to test models (Figure 2A). We compared differentially expressed genes (DEGs) between hi–77^–/–^ empty and hi–77^+/+^ empty, between hi–77^–/–^ empty and hi–77^–/–^ GATA2, and between hi–77^–/–^ empty and hi–77^–/–^ 9aa-Ins, and amalgamated data from 4 biological replicates quantified by RSEM (59). The DEGs (|log_2_(fold change)| ≥1 and adjusted *P* value < 0.05) were parsed into: –77-regulated, 2,084 DEGs; HA-GATA2–regulated, 2,138 DEGs; 9aa-Ins–regulated, 939 DEGs (Figure 2B). Analysis of gene expression changes (|log_2_(fold change)| > 0 and no adjusted *P* value cutoff) resulting from –77 deletion and HA-GATA2 expression in the rescue assay demonstrated that HA-GATA2 rescued 66% (4,768 of 7,258) and 68% (4,541 of 6,724) of genes activated and repressed, respectively, by endogenous GATA2 (Supplemental Figure 2A). There was greater overlap between –77- and HA-GATA2–regulated genes versus –77- and 9aa-Ins–regulated genes (916 vs. 377).

–77-, HA-GATA2–, and 9aa-Ins–regulated genes were parsed into activated and repressed (Figure 2B). The analysis revealed 2,138 GATA2-regulated genes, 525 GATA2- and 9aa-Ins–regulated genes, and 414 genes solely regulated by 9aa-Ins (ectopic) (Figure 2B). As GATA2 and 9aa-Ins regulated 525 genes, 9aa-Ins retained activity to regulate a minority of GATA2-regulated genes (Supplemental Table 1). To identify 9aa-Ins–regulated genes, we excluded genes with low expression (transcripts per million [TPM] <1 in any replicate of hi–77^–/–^ GATA2 for activation; hi–77^–/–^ empty for repression) and compared fold change of hi–77^–/–^ empty versus hi–77^–/–^ GATA2 and hi–77^–/–^ empty versus hi–77^–/–^ 9aa-Ins (Figure 2C). Of 215 genes, 79 were activated and 136 were repressed at 65% and 56%, respectively, of the level conferred by GATA2 (Figure 2C and Supplemental Figure 2B). Principal component analysis (PCA) demonstrated high data reproducibility (Figure 2D).

Since ectopic transcription factor activities may be significant (Figure 2A, model 4; and Supplemental Table 2), we tested whether the ectopic activity at 414 genes versus normal GATA2 activity was detected with *P* values from 0.01 to 0.1. As 47% of ectopically activated genes were retained at *P* = 0.1, and 40% of ectopically repressed genes at *P* = 0.01 (Figure 2E), ectopically 9aa-Ins–regulated genes emerged irrespective of stringency. Although GATA2-dependent repression is not understood, a similar number of genes were GATA2-activated (1,061 genes) and -repressed (1,077 genes), resembling GATA1 in erythroid cells (60, 61). GATA1-regulated genes, e.g., heme biosynthetic enzymes, hemoglobin subunits, and cytoskeletal components (16, 61–66), were not GATA2-regulated. One hundred forty-four of the 1,061 GATA2-activated genes (14%) were 9aa-Ins–activated, and 381 of 1,077 GATA2-repressed genes (35%) were 9aa-Ins–repressed. Analysis with *P* values from 0.01 to 0.1 revealed that the insertion impaired a greater percentage of activated versus repressed genes (86.4% of GATA2-activated genes were not 9aa-Ins–activated; 64.6% of GATA2-repressed genes were not 9aa-Ins–repressed; *P* = 9.1 × 10^–5^) (Figure 2F). Excluding genes with less than 1 TPM yielded identical conclusions (Supplemental Figure 2, C and D). Thus, without altering protein level and nuclear localization, zinc finger dislocation generated overt defects genome-wide.

### Inter–zinc finger spacing constraints for activation versus repression.

**Figure 3:**
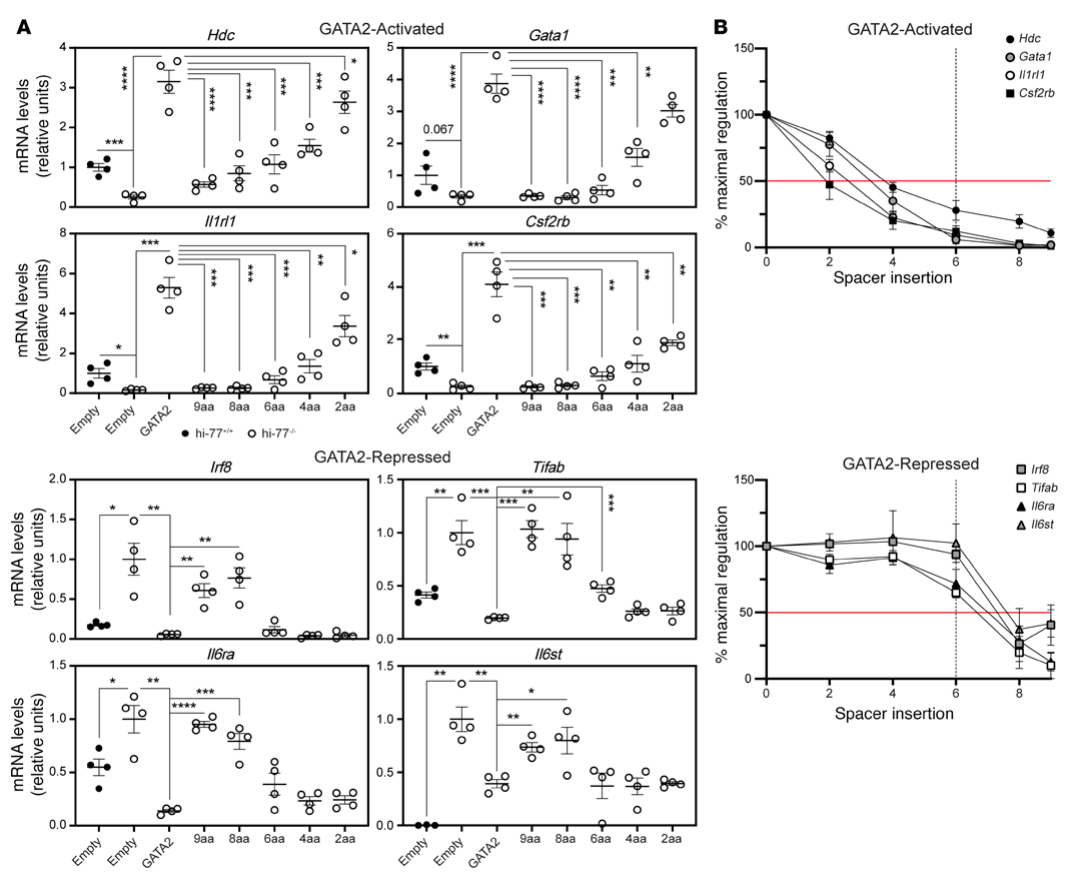
Inter–zinc finger spacer constraints for GATA2-mediated activation versus repression. (**A**) qRT-PCR analysis of mRNA expression in hi–77^–/–^ cells rescued with GATA2 or variants (*n* = 4). (**B**) Comparison of the percentage maximal activation and repression calculated with data from **A**. 100% or 0% activation and repression determined by analysis of hi–77^–/–^ cells with or without HA-GATA2. Red line, 50% regulation. Dotted line, 6aa insertion that impaired activation, but not repression, by more than 50%. Error bars represent mean ± SEM. Statistical calculations used unpaired 2-tailed Student’s *t* test with Benjamini-Hochberg correction; *P < 0.05; **P < 0.01; ***P < 0.001; ****P < 0.0001.

To establish whether the 9aa insertion is uniquely deleterious or whether smaller spacing alterations are inhibitory, we quantified expression of GATA2-activated (*Hdc*, *Gata1*, *Il1rl1*, *Csf2rb*) or -repressed (*Irf8*, *Tifab*, *Il6ra*, *Il6st*) genes detected by RNA-Seq (Figure 3A). Using the rescue assay, we compared activities of GATA2 and 2, 4, 6, 8, and 9aa variants to activate and repress transcription. Quantitative reverse transcriptase PCR (qRT-PCR) analysis with GATA2-expressing hi–77^–/–^ cells confirmed *Hdc*, *Gata1*, *Il1rl1*, and *Csf2rb* activation and *Irf8*, *Tifab*, *Il6ra*, and *Il6st* repression. 9aa-Ins was largely defective. Whereas the 8, 6, and 4aa variants were defective in activating *Hdc*, *Gata1*, *Il1rl1*, and *Csf2rb* expression, the 2aa variant was active. The 6, 4, and 2aa variants shared GATA2 activity to repress *Irf8*, *Tifab*, *Il6ra*, and *Il6st*, differing from 8aa, which resembled the defective 9aa-Ins. Although 6aa reduced GATA2-mediated activation by more than 50%, it repressed GATA2 target genes by at least 50% (Figure 3B). GATA2-mediated activation was reduced by insertions ≥2aa, whereas repression tolerated ≤6aa insertions. These results illustrate how variants can disrupt certain molecular processes, while sparing others.

### Leveraging a GATA2 pathogenic variant to elucidate how GATA2 controls genome function.

**Figure 4:**
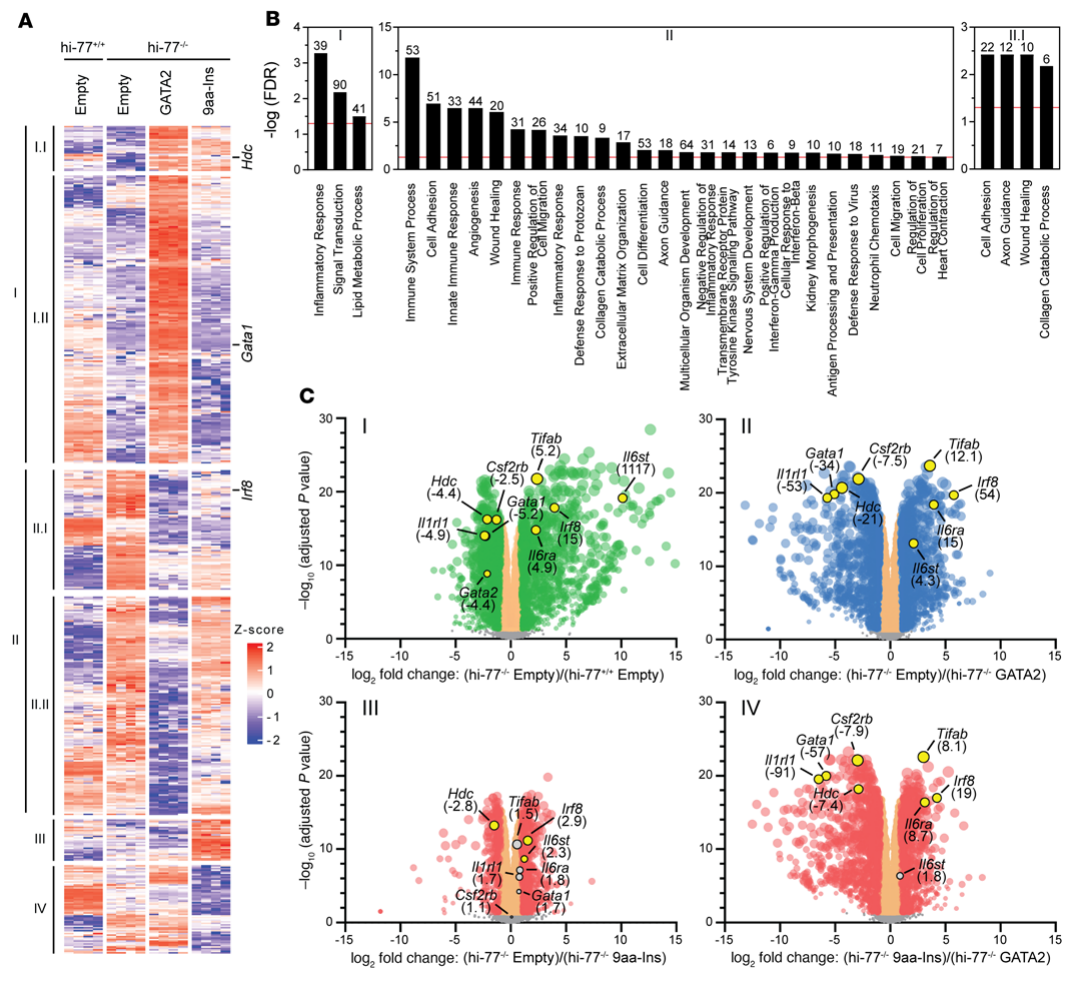
Biological/mechanistic insights revealed from *GATA2* disease variant transcriptomics. (**A**) DEGs that are GATA2-regulated, GATA2- and 9aa-Ins–regulated, and 9aa-Ins–regulated from comparison of hi–77^–/–^ empty (*n* = 4), hi–77^–/–^ GATA2 (*n* = 4), and hi–77^–/–^ 9aa-Ins (*n* = 4). *Z* score was calculated from each gene’s log_10_(FPKM+10^–3^) from all RNA-Seq replicates. GATA2 or 9aa-Ins expression in hi–77^–/–^ cells parsed the DEGs into: I, GATA2-activated; I.I, GATA2- and 9aa-Ins–activated; I.II, only GATA2-activated; II, GATA2-repressed; II.I, GATA2- and 9aa-Ins–repressed; II.II, only GATA2-repressed; III, ectopically activated; IV, ectopically repressed. (**B**) Gene Ontology (GO) analysis on 1,061 GATA2-activated, 1,077 GATA2-repressed, and 381 GATA2- and 9aa-Ins–repressed genes. Bar graphs represent –log(FDR) with a red line at FDR < 0.05 to determine statistical significance. Significant GO terms are presented. The number of genes comprised by each term is shown above the graphs. (**C**) Plots I, II, and III, expression changes from –77, GATA2, and 9aa-Ins regulation. 2,084 enhancer-regulated, 2,138 GATA2-regulated, and 939 GATA2-regulated genes from **B** are color-coded in green, blue, and pink, respectively. Plot IV, magnitude of expression between GATA2 and 9aa-Ins. DEGs are depicted in pink. GATA2-activated and -repressed DEGs are highlighted. Fold change relative to hi–77^–/–^ empty or hi–77^–/–^ GATA2 is shown in parentheses.

9aa-Ins could not establish the GATA2-dependent transcriptome, yet it retained some capacity to regulate select GATA2 target genes (Figure 4A). To unveil pathways/networks important for hematopoiesis, we stratified DEGs from hi–77^–/–^ empty versus hi–77^–/–^ GATA2 and hi–77^–/–^ empty versus hi–77^–/–^ 9aa based on regulatory attributes and Gene Ontology to yield: I, GATA2-activated; I.I, GATA2- and 9aa-activated; I.II, only GATA2-activated; II, GATA2-repressed; II.I, GATA2- and 9aa-repressed; II.II, only GATA2-repressed; III, ectopically activated (9aa-Ins–activated but not GATA2-activated); IV, ectopically repressed (9aa-Ins–repressed but not GATA2-repressed) (Figure 4A). The 9aa insertion impaired GATA2-mediated activation of genes related to G protein–coupled receptor signaling (e.g., *Cnr2*, *S1pr1*, *S1pr4*) and repression of innate immune genes (e.g., *Irf8*, *Tlr1*, *Tlr6*, *Tlr7*, *Il7*) (Figure 4B and Supplemental Figure 3). 9aa-Ins– but not GATA2-regulated genes (ectopic) did not reveal mechanistic insights. 9aa-Ins retained some activity to repress GATA2-repressed genes, including *Tgfa*, *Vwf*, *Fn1*, and *Tgfb3*, which mediate cell adhesion, wound healing, axon guidance, and collagen catabolic process (Figure 4B and Supplemental Figure 3), as well as the B-lineage genes *Pax5*, *Rag1*, and *Rag2* (Supplemental Table 1).

–77 deletion reduced *Gata2* expression 4.4-fold. We identified –77-regulated genes by comparing transcriptomes of hi–77^–/–^ empty versus hi–77^+/+^ empty cells. –77 deletion revealed 960 –77-activated and 1,124 –77-repressed genes (Figure 2B and Figure 4C). Many –77-activated (*Hdc*, *Gata1*, *Il1rl1*, *Csf2rb*) and -repressed (*Irf8*, *Tifab*, *Il6ra*, *Il6st*) genes (Figure 4C, plot I) were GATA2-activated and -repressed upon rescue (Figure 4C, plot II). 9aa-Ins was impaired in activating and repressing these genes (Figure 4C, plot III), and at most genes, it was less active than GATA2 (Figure 4C, plot IV). These analyses established the GATA2 contribution to the progenitor transcriptome and extreme differences between GATA2 and 9aa-Ins.

**Figure 5:**
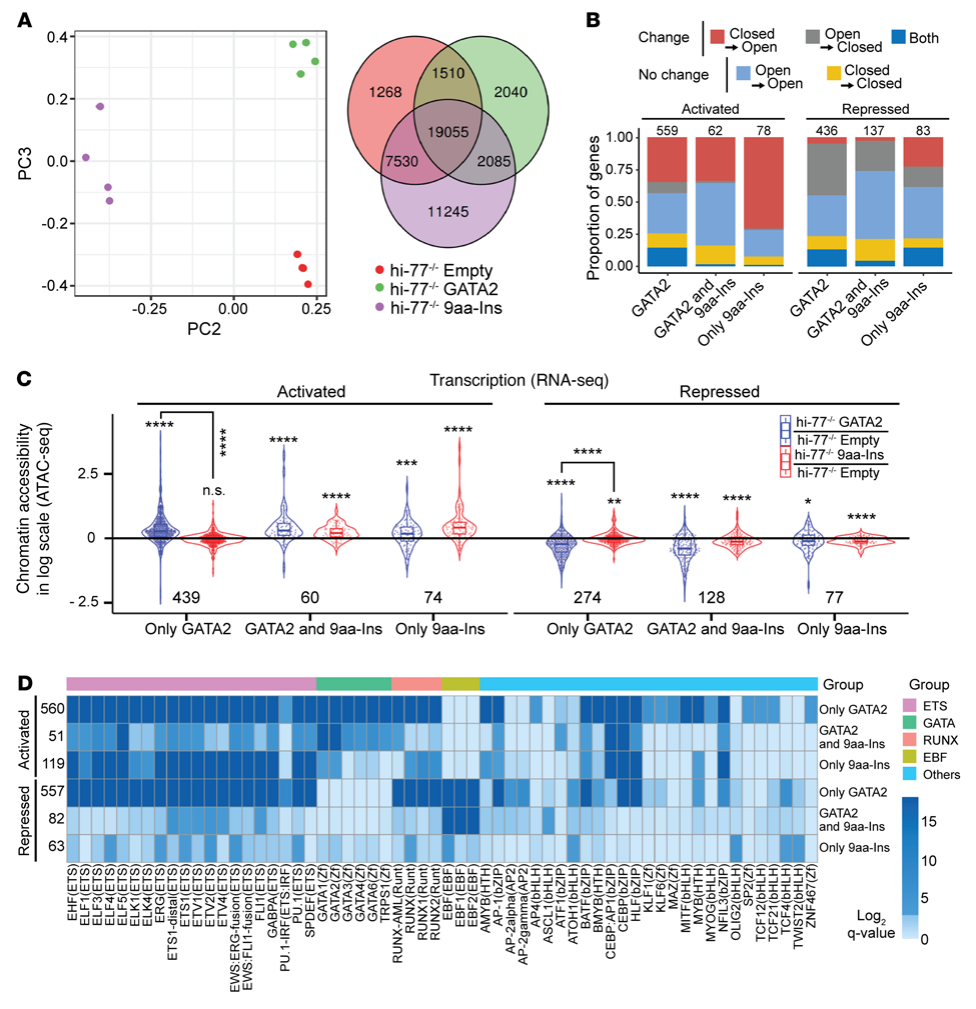
Multiomic analysis with GATA2 disease variant reveals principles of GATA2 function through chromatin. (**A**) PCA quantifying multidimensional scaling distances between differential chromatin accessibility (ATAC-Seq, GEO GSE201968) with *n* = 4 biological replicates of hi–77^–/–^ empty, hi–77^–/–^ GATA2, hi–77^–/–^ 9aa-Ins. Venn diagram depicts overlap. (**B**) Chromatin transitions of genes activated or repressed by GATA2, both GATA2 and 9aa-Ins, or only 9aa-Ins. Number of genes comprised by each group is shown above the graphs. (**C**) GATA2 and 9aa-Ins impact on chromatin accessibility. hi–77^–/–^ GATA2/hi–77^–/–^ empty signal or hi–77^–/–^ 9aa-Ins/hi–77^–/–^ empty signal was determined at genes activated/repressed by only GATA2, GATA2 and 9aa-Ins, and only 9aa-Ins conditions by amalgamation of ATAC-Seq peaks linked to DEGs. Statistical calculations to measure chromatin accessibility (>0 or <0 for differential accessibility) used Wilcoxon’s rank sum test. Comparisons between 2 groups used Wilcoxon’s signed rank test. *P < 0.05; **P < 0.01; ***P < 0.001; ****P < 0.0001. (**D**) Motif enrichment analysis at differentially accessible loci activated or repressed by only GATA2, GATA2 and 9aa-Ins, or only 9aa-Ins. Number of peaks comprised by each group is on the left of the heatmap. Peaks less than 100 kb from the start site were analyzed.

Since 9aa-Ins was compromised in activating and repressing many loci, ATAC-Seq (assay for transposase-accessible chromatin using sequencing) was used to ask whether 9aa-Ins is defective in remodeling of chromatin or in steps after chromatin regulation. Integrating ATAC-Seq and RNA-Seq data linked differentially accessible peaks to DEGs. Peaks were called in the 4 replicates by MACS2 (Model-Based Analysis of ChIP-Seq), and Irreproducible Discovery Rate (IDR) was used to identify 44,733 reproducible (master) peaks. PCA of peaks across the samples confirmed reproducibility and definitive separation among the samples (Figure 5A). Using DESeq2, differentially accessible ATAC-Seq peaks were determined by amalgamation of overlapping peaks from the replicates of hi–77^–/–^ empty, hi–77^–/–^ GATA2, and hi–77^–/–^ 9aa-Ins. Differentially accessible peaks in hi–77^–/–^ GATA2 and hi–77^–/–^ 9aa-Ins were defined as |log_2_(fold change)| > 1 and adjusted *P* value < 0.05 relative to hi–77^–/–^ empty.

To test whether GATA2-mediated activation and repression similarly involve chromatin remodeling, we analyzed ATAC-Seq peaks at GATA2-regulated genes by accessibility and magnitude of changes. At GATA2-activated and -repressed loci, we parsed differential peaks by accessibility and assigned them to the nearest DEG (Supplemental Methods). Analyzing differentially regulated peaks in hi–77^–/–^ GATA2/hi–77^–/–^ empty revealed peaks linked to 559 GATA2-activated and 436 GATA2-repressed DEGs (Figure 5B). GATA2-mediated activation (559 genes) more frequently involved chromatin opening versus closing (35% versus 8.6%), while GATA2-mediated repression (436 genes) more frequently involved closing versus opening (40% versus 5.0%). The proportion of GATA2-activated genes with opening was comparable to that of GATA2-repressed genes with closing (35% versus 40%).

To compare GATA2 and 9aa-Ins activities to remodel chromatin, we quantified chromatin changes at 1,613 DEGs that were exclusively GATA2-regulated (917 activated and 696 repressed) (Figure 2B). Normalized ATAC-Seq peak signals from 439 genes activated by GATA2, but not 9aa-Ins, increased significantly (Figure 5C; Wilcoxon’s rank sum test *P* value < 2.1 × 10^–41^). Normalized peak signals from 274 genes repressed by GATA2, but not 9aa-Ins, decreased significantly (Wilcoxon’s rank sum test *P* < 2.0 × 10^–23^). The analysis at promoters (–2 kb to +100 bp) yielded similar conclusions (Supplemental Figure 4A).

As 9aa-Ins was defective in activating and repressing most GATA2-regulated genes, we asked whether 9aa-Ins failed to remodel chromatin. Analyzing differentially accessible peaks in hi–77^–/–^ GATA2/hi–77^–/–^ empty and hi–77^–/–^ 9aa-Ins/hi–77^–/–^ empty revealed 62 GATA2- and 9aa-Ins–activated and 137 GATA2- and 9aa-Ins–repressed genes (Figure 5B). The proportion of 21 mutually activated genes with chromatin opening was only 3.8% of 559 GATA2-activated genes. Thirty-two mutually repressed genes with chromatin closing constituted 7.3% of 436 GATA2-repressed genes. The 21 mutually activated genes with chromatin opening and 32 mutually repressed genes with chromatin closing represented 34% and 23% within their cohort of mutually regulated genes. Comparison of GATA2-activated with mutually activated genes revealed a similar proportion of genes (35% vs. 34%) with chromatin opening (proportion test *P* < 0.08193). Comparison of GATA2-repressed with mutually repressed genes revealed a decreased proportion of genes (40% vs. 23%) with chromatin closing (proportion test *P* < 1.495 × 10^–6^). 9aa-Ins retained activity at a minority of loci.

To quantify chromatin changes, we analyzed 525 DEGs (144 activated and 381 repressed) that were GATA2- and 9aa-Ins–regulated (Figure 2B). Sixty activated and 128 repressed genes harbored differential peaks. ATAC-Seq data from 60 GATA2- and 9aa-Ins–activated genes revealed chromatin opening by GATA2 (*P* < 8.737 × 10^–9^) and 9aa-Ins (*P* < 7.502 × 10^–8^), while ATAC-Seq data from 128 GATA2- and 9aa-Ins–repressed genes revealed chromatin closing by GATA2 (*P* < 8.875 × 10^–15^) and 9aa-Ins (*P* < 1.337 × 10^–7^). Peak signals from promoters yielded similar conclusions (Supplemental Figure 4A). Since GATA2 and 9aa-Ins remodeled chromatin similarly at mutually regulated genes, we assessed whether 9aa-Ins defect in transcriptional regulation is linked to its inability to open or close chromatin at genes regulated by GATA2, but not 9aa-Ins. We compared hi–77^–/–^ GATA2/hi–77^–/–^ empty versus hi–77^–/–^ 9aa-Ins/hi–77^–/–^ empty ATAC-Seq data from 439 exclusively GATA2-activated and 274 exclusively GATA2-repressed genes (Figure 5C). In 439 exclusively GATA2-activated genes, 9aa-Ins–mediated chromatin opening was attenuated relative to GATA2 (*P* < 3.031 × 10^–5^). In 274 exclusively GATA2-repressed genes, 9aa-Ins–mediated chromatin closing was attenuated (*P* < 4.081 × 10^–10^) (Figure 5C). Similar conclusions emerged from promoter analyses (Supplemental Figure 4A). 9aa-Ins was defective in regulating chromatin at genes exclusively regulated by GATA2.

 To test whether 9aa-Ins regulates chromatin at ectopic loci, ATAC-Seq peaks were linked to 414 ectopically regulated genes (132 activated, 282 repressed) (Figure 2B). ATAC-Seq analysis with hi–77^–/–^ 9aa-Ins/hi–77^–/–^ empty, but not hi–77^–/–^ GATA2/hi–77^–/–^ empty, revealed 78 ectopically activated and 83 ectopically repressed genes (Figure 5B). Fifty-five of 78 genes (70%) that were only activated by 9aa-Ins exhibited chromatin opening, and 13 of the 83 genes (16%) only repressed by 9aa-Ins exhibited chromatin closing. Analysis of 74 ectopically activated genes revealed increased accessibility (*P* < 7.502 × 10^–8^), while peaks at 77 ectopically repressed genes revealed reduced accessibility (*P* < 1.337 × 10^–7^) (Figure 5C). At select loci in which 9aa-Ins uniquely controlled transcription, it ectopically regulated chromatin.

Motif analysis was conducted with 560 and 557 differentially accessible peaks linked to GATA2-activated and -repressed genes, respectively (not 9aa-Ins–regulated). The GATA2-binding WGATAR motif was enriched in ATAC-Seq peaks at activated loci (Figure 5D) and underrepresented in peaks from repressed loci (Figure 5D), which was unpredictable since GATA2-dependent repression mechanisms are not established. De novo motif finding from enrichment (Supplemental Figure 4B) and discriminative (Supplemental Figure 4C) analyses yielded similar results. ETS motifs were enriched at sites within GATA2-repressed loci (Figure 5D) to a greater extent than GATA2-activated loci (Supplemental Figure 4C).

As WGATAR was enriched at GATA2-activated loci, we asked whether E-box–spacer–WGATAR composite elements and double WGATAR motifs were also enriched. We identified sequences with 6- to 14-bp spacers between E-box and WGATAR and up to 5 bp spacer between 2 WGATARs. Composite elements resided at 11.3% and 6.46% and double WGATAR motifs at 1.25% and 0.72% of ATAC-Seq peaks at GATA2-activated and -repressed loci, respectively.

Given the ETS motif enrichment at GATA2-repressed loci, and ETS protein activation functions (67), we tested whether GATA2 downregulates genes encoding ETS factors. While 15 of 26 ETS factors (*Erg*, *Etv3*, *Etv4*, *Etv5*, *Etv6*, *Elf1*, *Elf2*, *Elf4*, *Elk1*, *Elk3*, *Elk4*, *Ets2*, *Gabpa*, *Spi1*, *Fli1*) were expressed (TPM ≥1 in all conditions) in progenitors, GATA2 and 9aa-Ins did not alter their expression (Supplemental Table 3).

9aa-Ins regulated chromatin accessibility and activated or repressed a minority of GATA2-regulated genes (Figure 5B and Supplemental Table 1). Fifty-one differentially accessible ATAC-Seq peaks at GATA2- and 9aa-Ins–activated genes harbored fewer WGATAR motifs (Figure 5D) versus only GATA2-activated genes (Supplemental Figure 4C). Eighty-two differentially accessible peaks at GATA2- and 9aa-Ins–repressed genes harbored significantly fewer ETS motifs versus only GATA2-repressed loci (Figure 5D and Supplemental Figure 4C). Comparing motifs at loci regulated by exclusively GATA2, GATA2 and 9aa-Ins, or exclusively 9aa-Ins, enriched motifs (Figure 5D) were not detected from discriminative analyses (Supplemental Figure 4C) of 119 differentially accessible peaks at ectopically 9aa-Ins–activated genes and 63 differentially accessible peaks at ectopically 9aa-Ins–repressed loci.

To test whether defective transcriptional and chromatin regulation by 9aa-Ins involves impaired DNA binding, we generated recombinant GATA2 and 9aa-Ins double–zinc finger proteins in *E.* *coli*, purified to about 80% purity (Supplemental Figure 5A), and conducted electrophoretic mobility shift assay (EMSA). One-dimensional ^1^H NMR spectra detected comparable folding between WT and 9aa-Ins, with additional signals representing the inserted 9 aa residues; the zinc fingers were not destabilized (Supplemental Figure 5B). EMSA with increasing protein concentrations and probes harboring single WGATAR sequences (AGATAA or TGATAA) of the GATA2-activated *Kit* locus (–114 kb) or double GATA motif (GGATAAAGATC) (68) revealed that 9aa-Ins zinc fingers had reduced DNA binding capacity with TGATAA and double GATA probes. WT GATA2 fingers did not stably bind the AGATAA probe; although the oligonucleotide was bound, stable complexes were unresolved (Supplemental Figure 5C).

To establish whether the 9aa insertion affects chromatin occupancy, we conducted CUT&Tag with anti-HA antibody in hi–77^–/–^ cells expressing GATA2 or 9aa-Ins. Peaks were called with MACS3 and merged with HOMER mergePeaks (69) and Diffbind (70) (6,014 GATA2 and 1,051 9aa-Ins peaks; Supplemental Figure 6A). Linking CUT&Tag peaks to the nearest GATA2-regulated genes from RNA-Seq yielded 561 GATA2 and 35 9aa-Ins peaks at activated loci and 190 GATA2 and 27 9aa-Ins peaks at repressed loci (Supplemental Figure 6B). As only 33 GATA2 and 9aa-Ins peaks at activated loci and 20 peaks at repressed loci were detected, the 9aa insertion reduced occupancy at 94% (528 of 561) and 90% (170 of 190) of activated and repressed loci, respectively. ChIP-qPCR with hi–77^–/–^ cells expressing comparable HA-GATA2 and 9aa-Ins levels (Supplemental Figure 6C) confirmed the loss of 9aa-Ins occupancy (*Gata1* and *Hdc* promoters, *Kit* –114) (Supplemental Figure 6D). Comparison of activated versus repressed loci revealed a 3.0-fold increase in GATA2 occupancy sites at activated loci (561 vs. 190) (Supplemental Figure 6B), despite a similar WGATAR distribution at activated and repressed loci (363 of 561 peaks [69%] at activated loci and 104 of 190 peaks [61%] at repressed loci; 2-sample test for equality of proportions with continuity correction, *P* = 0.08).

**Figure 6:**
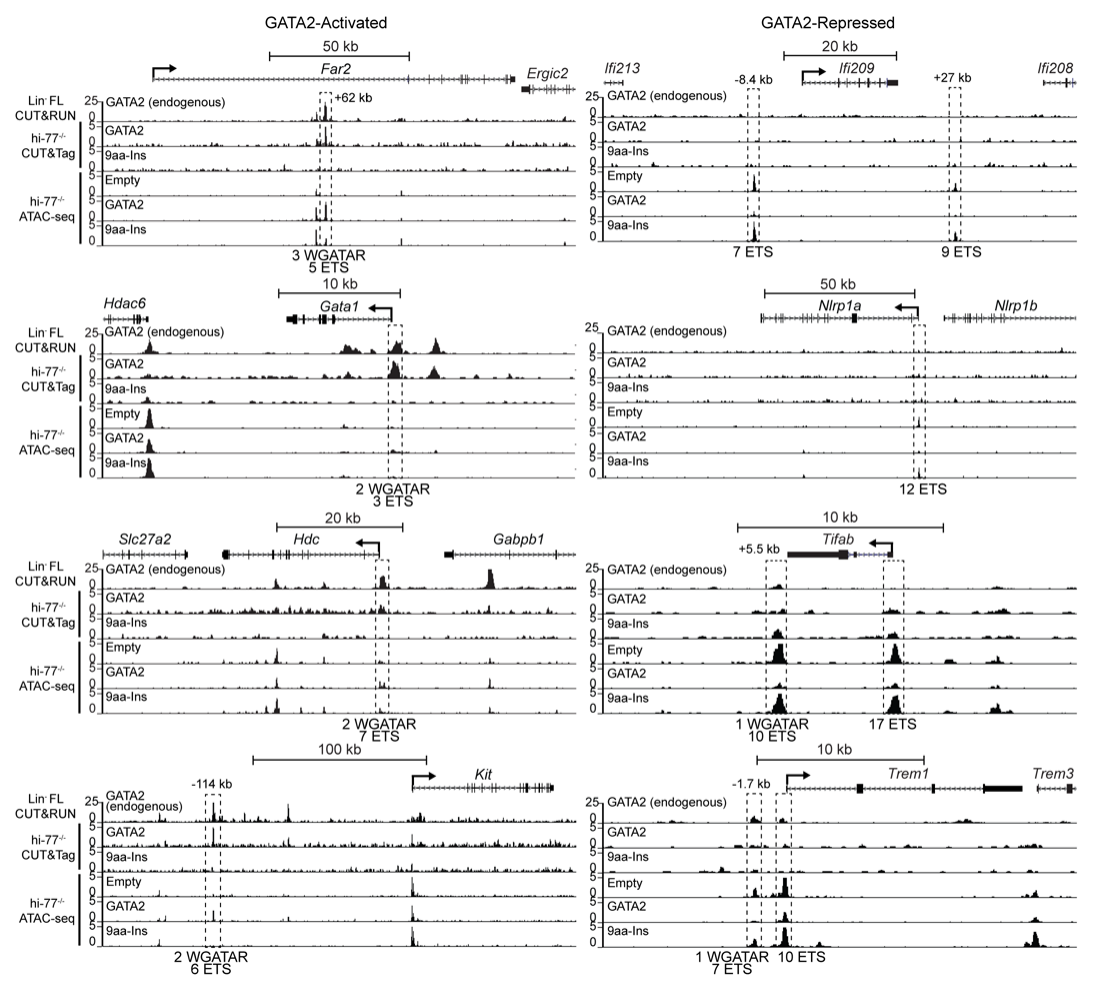
GATA2 occupancy and GATA2-regulated chromatin remodeling at GATA2-activated and -repressed loci. GATA2 CUT&RUN with fetal liver Lin^–^ erythroid progenitors (Lin^–^ FL) and HA CUT&Tag with hi–77^–/–^ GATA2 and hi–77^–/–^ 9aa-Ins revealed GATA2 occupancy at GATA2-activated but not -repressed loci corresponding to ATAC-Seq profiles of GATA2-activated and GATA2-repressed genes. Peaks near the genes are boxed by dashed lines. WGATAR and ETS motifs located at peak sites are tabulated below.

At loci in which GATA2 remodeled chromatin, GATA2 occupied the GATA2-activated loci *Far2*, *Gata1*, *Hdc*, and *Kit* with differentially accessible ATAC-Seq peaks in hi–77^–/–^ GATA2/hi–77^–/–^ empty (Figure 6). GATA2 did not occupy the GATA2-repressed loci *Ifi209*, *Tifab*, *Nlrp1a*, and *Trem1* with ATAC-Seq peaks that were less accessible in hi–77^–/–^ GATA2/hi–77^–/–^ empty (Figure 6). 9aa-Ins lacked occupancy at these loci (Figure 6). The lack of chromatin occupancy at select repressed loci suggests that GATA2-regulated genome repression also occurs indirectly.

### GATA2 suppresses a lineage-diverting gene expression program.

**Figure 7:**
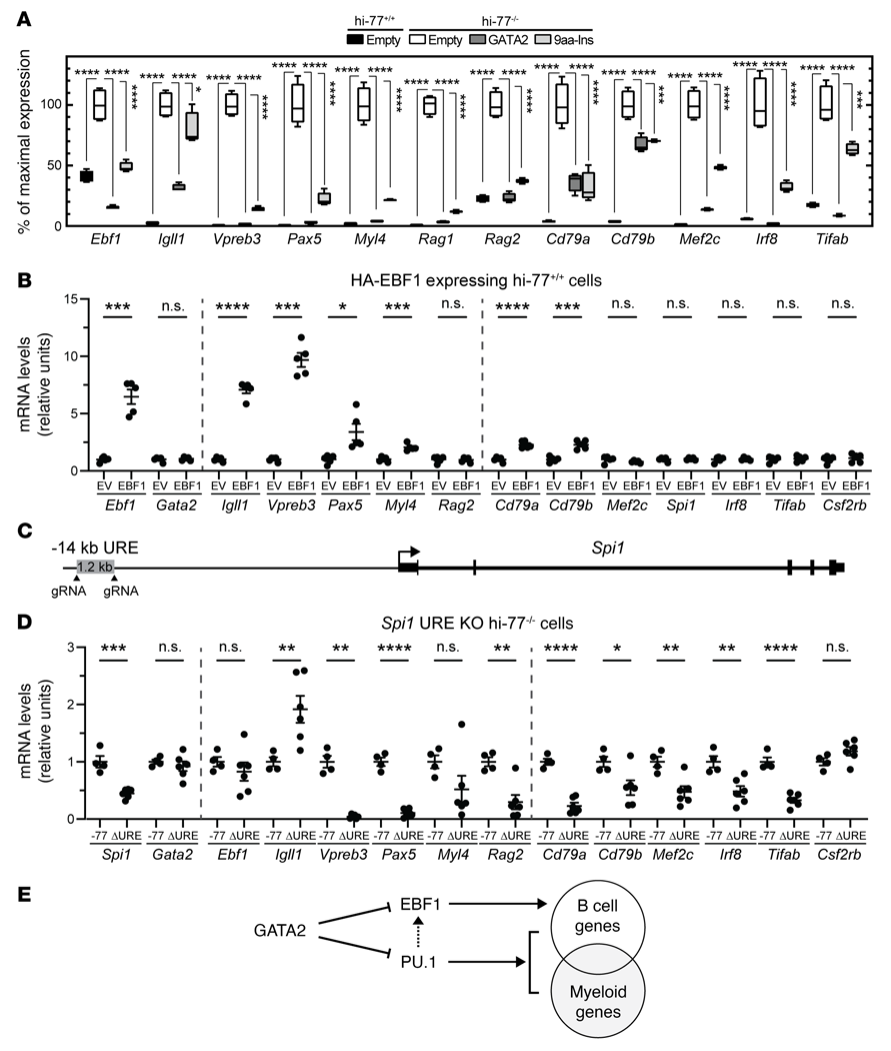
GATA2 opposes a B-lineage gene expression program. (**A**) Expression of GATA2-repressed CLP and B-lineage or myeloid genes in hi–77^+/+^ empty, hi–77^–/–^ empty, hi–77^–/–^ GATA2, and hi–77^–/–^ 9aa-Ins from the RNA-Seq of Figure 2. Average TPM of hi–77^–/–^ empty is presented as 100% of maximal expression. For multiple comparisons with hi–77^–/–^ empty control, statistics were calculated using 1-way ANOVA followed by Dunnett’s test; *P < 0.05; ***P < 0.001; ****P < 0.0001. (**B**) mRNA levels of GATA2-repressed genes in hi–77^+/+^ cells infected with retrovirus to express EBF1 or empty vector (EV). Error bars represent mean **±** SEM. Statistical calculations used unpaired 2-tailed Student’s *t* test. Welch’s correction was applied when variances were unequal (*Ebf1*, *Igll1*, *Vpreb3*, *Pax5*); *P < 0.05; ***P < 0.001; ****P < 0.0001. (**C**) Targeted ablation of a *Spi1* (encoding PU.1) enhancer –14 kb upstream regulatory element (ΔURE) (77, 78). (**D**) GATA2-repressed target gene mRNA levels in hi–77^–/–^ versus hi–77^–/–^ cells lacking PU.1 enhancer. Error bars represent mean **±** SEM. Statistical calculations used unpaired 2-tailed Student’s *t* test. Welch’s correction was applied when variances were unequal (*Vpreb3*); *P < 0.05; **P < 0.01; ***P < 0.001; ****P < 0.0001. (**E**) GATA2-mediated repression with GATA2 occupying and repressing *Ebf1* expression and opposing PU.1-mediated activation. PU.1 induction of *Ebf1* expression (75, 76, 123) is indicated by a dashed line.

To elucidate mechanisms underlying GATA2-mediated repression, we evaluated motifs enriched at GATA2-repressed loci. Motifs for the B-lineage developmental regulator EBF1 (38, 71, 72) were enriched at repressed loci (Figure 5D and Supplemental Figure 4C). *Ebf1* expression was low (7.5 TPM) in hi–77^+/+^ cells, as expected for a lymphopoiesis-driving gene. *Ebf1* expression was upregulated 2.5-fold in hi–77^–/–^ cells (*P* = 0.0002) (Figure 7A) and 4.0-fold in -77^–/–^ primary fetal liver progenitors (*P* = 0.028) (55). GATA2 expression in the rescue assay reduced *Ebf1* expression by 84% (*P* < 0.0001) (Figure 7A). The genes upregulated in hi–77^–/–^ cells and repressed by GATA2 included a cohort with expression enriched in B-lineage cells (haemosphere.org) (*Ebf1*, *Igll1*, *Vpreb3*, *Pax5*, *Myl4*, *Rag1*, and *Rag2*), which are vital for B-lineage biology and B-lineage/myeloid genes (*Cd79a*, *Cd79b*, *Mef2c*, and *Irf8*) (Figure 7A). GATA2 occupied the *Ebf1* promoter and remodeled chromatin (Supplemental Figure 7). In pre–B cells, EBF1 occupied GATA2-regulated ATAC-Seq peaks at *Igll1*, *Vpreb3*, *Pax5*, *Cd79a*, and *Cd79b* (Supplemental Figure 7). To test whether elevated EBF1 suffices to activate B-lineage genes in progenitors, we expressed HA-EBF1 in hi–77^+/+^ cells. *Ebf1* transcripts were 6.5-fold higher in HA-EBF1–expressing versus control (empty vector) cells. *Gata2* transcripts were unchanged. HA-EBF1 increased expression of B-lineage (*Igll1*, *Vpreb3*, *Pax5*, *Myl4*) and B-lineage/myeloid genes (*Cd79a* and *Cd79b*) (Figure 7B).

PU.1 regulates B-lineage and myeloid genes and binds ETS motifs that exist at GATA2-repressed loci (Figure 5D and Supplemental Figure 4C). GATA2-PU.1 antagonism can determine myeloid fate (73–75), and GATA2 loss does not alter PU.1 levels (55). At loci in which GATA2 remodeled chromatin, PU.1 occupied *Ebf1*, *Vpreb3*, *Pax5*, *Myl4*, *Cd79a*, *Cd79b*, *Mef2c*, *Irf8*, *Tifab*, and *Csf2rb* (Supplemental Figure 7). To determine whether downregulating PU.1 in hi–77^–/–^ cells reduces expression of B-lineage genes upregulated in GATA2-deficient progenitors, we ablated the –14 kb upstream regulatory element (URE) from *Spi1* (encoding PU.1) in hi–77^–/–^ cells (76–78) (Figure 7C). *Spi1* expression decreased 2.2-fold in ΔURE cells versus hi–77^–/–^ without affecting *Gata2* expression. GATA2-repressed B-lineage genes (*Vpreb3*, *Pax5*, *Rag2*, *Cd79a*, *Cd79b*, *Mef2c*, *Irf8*, and *Tifab*) were downregulated in the ΔURE cells (Figure 7D). *Csf2rb*, a GATA2-activated gene (Figure 4C), was unaffected (Figure 7D). These results support a model in which GATA2 represses *Ebf1* expression and antagonizes PU.1 in fetal progenitors to suppress a B-lineage expression program that is discordant with myeloerythroid differentiation (Figure 7E).

### GATA2 deficiency establishes a hematopoiesis-disrupting cytokine signaling network.

**Figure 8:**
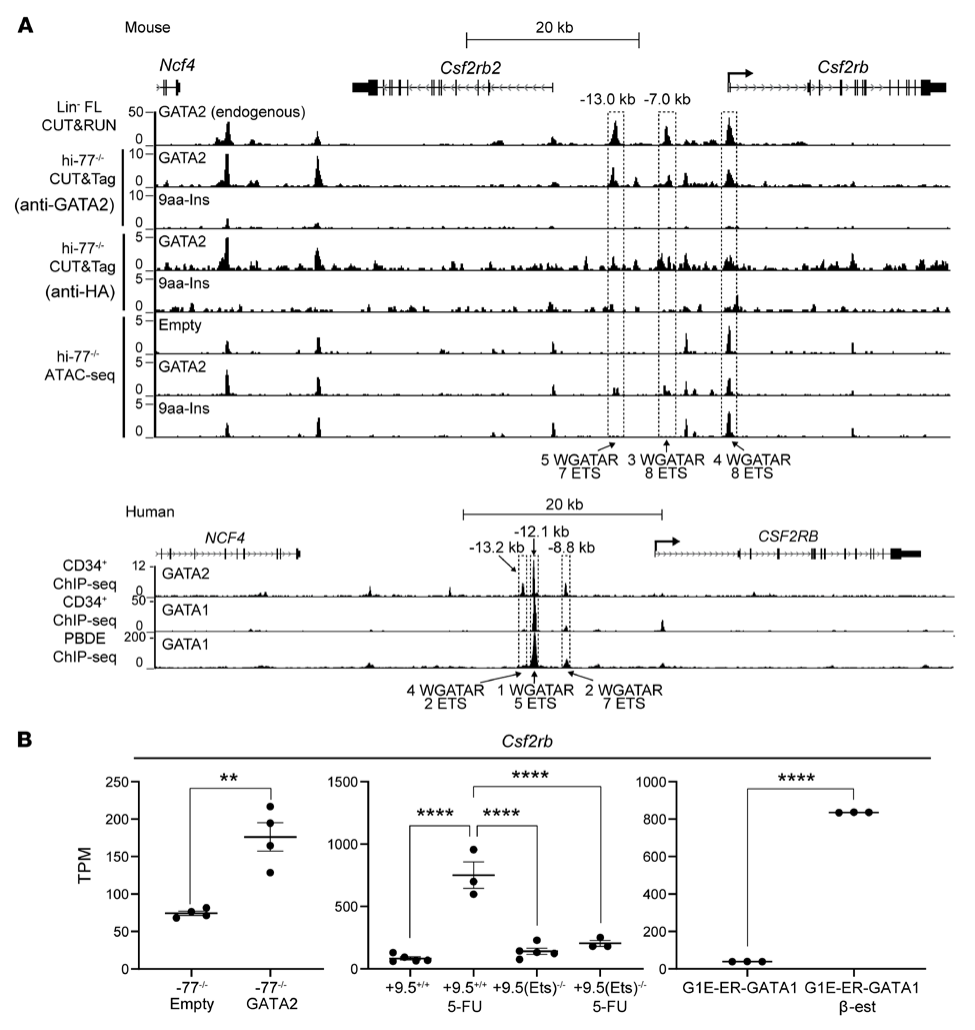
GATA1 and GATA2 occupy and remodel *Csf2rb* chromatin. (**A**) In mice, GATA2 CUT&RUN with Lin^–^ FL and GATA2 or HA CUT&Tag with hi–77^–/–^ GATA2 and hi–77^–/–^ 9aa-Ins revealed GATA2, but not 9aa-Ins, occupancy at the start site and 7 kb and 13 kb upstream of *Csf2rb*. ATAC-Seq revealed sites 7 kb and 13 kb upstream that were accessible only in hi–77^–/–^ GATA2 cells. ChIP-Seq with human CD34^+^ cells (78) revealed GATA2 occupancy 8.8, 12.1, and 13.2 kb upstream of *CSF2RB*. GATA1 ChIP-Seq with CD34^+^ cells (79) and peripheral blood–derived erythroblast (PBDE) cells (79) revealed GATA1 occupancy 8.8 and 12.1 kb upstream. (**B**) GATA2 regulation of *Csf2rb* (RNA-Seq). GATA2-mediated activation of *Csf2rb* in primary Lin^–^ –77^–/–^ progenitors with or without GATA2 (–77^–/–^ empty vs. –77^–/–^ GATA2) (61), WT or 9.5(Ets) motif–mutant bone marrow LSK cells with 5-FU [9.5^+/+^ 5-FU vs. 9.5(Ets)^–/–^ 5-FU] (82), and G1E-ER-GATA1 erythroblasts with or without β-estradiol (G1E-ER-GATA1 vs. G1E-ER-GATA1 β-est) (68). To compare differences between 2 groups, statistical calculations used unpaired 2-tailed Student’s *t* test. For multiple comparisons, unpaired 1-way ANOVA was used, followed by Tukey’s test; **P < 0.01; ****P < 0.0001.

GATA2 regulated genes encoding signaling proteins that were not 9aa-Ins–regulated (Supplemental Table 4). These genes included GATA2-activated *Csf2rb* (Figure 4C), encoding the shared common β chain of GM-CSF, IL-3, and IL-5 receptors (39, 40, 79). Since reduced GM-CSF signaling causes pulmonary alveolar proteinosis, a GATA2 deficiency syndrome phenotype (44, 45), we analyzed the underlying mechanism. To test whether GATA2 occupies *Csf2rb*, we assessed GATA2 CUT&RUN peaks in fetal liver Lin^–^ progenitors (Lin^–^ FL), GATA2 and HA CUT&Tag peaks in hi–77^–/–^ GATA2 and hi–77^–/–^ 9aa-Ins, ATAC-Seq peaks in hi–77^–/–^ GATA2/hi–77^–/–^ empty, and ChIP-Seq from human CD34^+^ and peripheral blood–derived erythroblast cells (80–82). GATA1 and GATA2 occupied sites upstream of *Csf2rb* (Figure 8A) and regulated chromatin accessibility. Comparing WT versus GATA2-deficient conditions (55, 83) or G1E-ER-GATA1 erythroblasts (60) revealed GATA2 and GATA1 induction of *Csf2rb* expression (Figure 8B).

**Figure 9:**
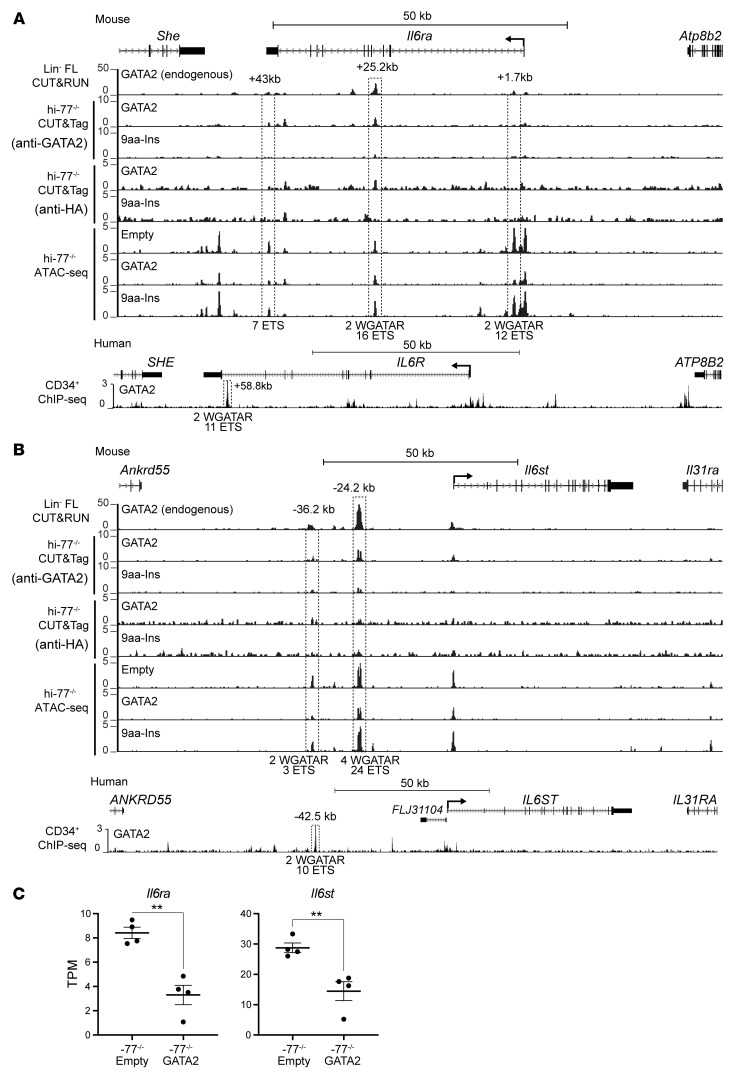
GATA2 occupies and remodels chromatin at *Il6ra* and *Il6st*. (**A**) GATA2 CUT&RUN with murine Lin^–^ FL and GATA2 or HA CUT&Tag with hi–77^–/–^ GATA2 and hi–77^–/–^ 9aa-Ins revealed GATA2, but not 9aa-Ins, occupancy 25.2 kb downstream of *Il6ra*. ATAC-Seq revealed intronic (1.7 kb downstream) and 3′-UTR (43 kb downstream) sites less accessible in hi–77^–/–^ GATA2 cells. ChIP-Seq with human CD34^+^ cells (78) revealed GATA2 occupancy 58.8 kb downstream of *IL6R*. (**B**) GATA2 ChIP-Seq with murine Lin^–^ fetal liver and GATA2 CUT&Tag with hi–77^–/–^ GATA2 and hi–77^–/–^ 9aa-Ins revealed GATA2, but not 9aa-Ins, occupancy 24.2 kb upstream of *Il6st*. ATAC-Seq with hi–77^–/–^ empty, GATA2, and 9aa-Ins revealed intergenic sites (24.2 kb and 36.2 kb upstream) less accessible in hi–77^–/–^ GATA2 cells. ChIP-Seq with human CD34^+^ cells (78) revealed occupancy 42.5 kb upstream of *IL6ST*. (**C**) GATA2-mediated repression of *Il6ra* and *Il6st* in primary Lin^–^ –77^–/–^ hematopoietic progenitors with and without GATA2 (–77^–/–^ empty vs. –77^–/–^ GATA2) (55). Statistical calculations used unpaired 2-tailed Student’s *t* test; **P < 0.01.

By contrast to reduced *Csf2rb* expression in GATA2-deficient progenitors, genes encoding IL-6 receptor α and β subunits (*Il6ra* and *Il6st*) were upregulated due to impaired GATA2-mediated repression (Figure 4C). *Il6st* encodes GP130, a receptor subunit for IL-6 family members (IL-6, IL-11, and IL-27 among others) (49). Elevated IL-6 in acute myeloid leukemia (AML) induces bone marrow failure (49, 84) and promotes myelodysplastic syndrome (MDS) to AML progression (50). CUT&RUN peaks in Lin^–^ progenitors, GATA2 and HA CUT&Tag peaks in hi–77^–/–^ GATA2 and hi–77^–/–^ 9aa-Ins, and differentially accessible ATAC-Seq peaks in hi–77^–/–^ GATA2/hi–77^–/–^ empty revealed GATA2 occupancy and regulated chromatin at *Il6ra* intronic sites and 3′-UTR, and *Il6st* upstream sites (Figure 9, A and B). GATA2 ChIP-Seq with CD34^+^ cells (79) revealed intronic and upstream peaks for *Il6ra* and *Il6st*, respectively (Figure 9, A and B). *Il6ra* and *Il6st* mRNA levels were upregulated in GATA2-deficient immortalized and primary progenitors (Figure 9C).

**Figure 10:**
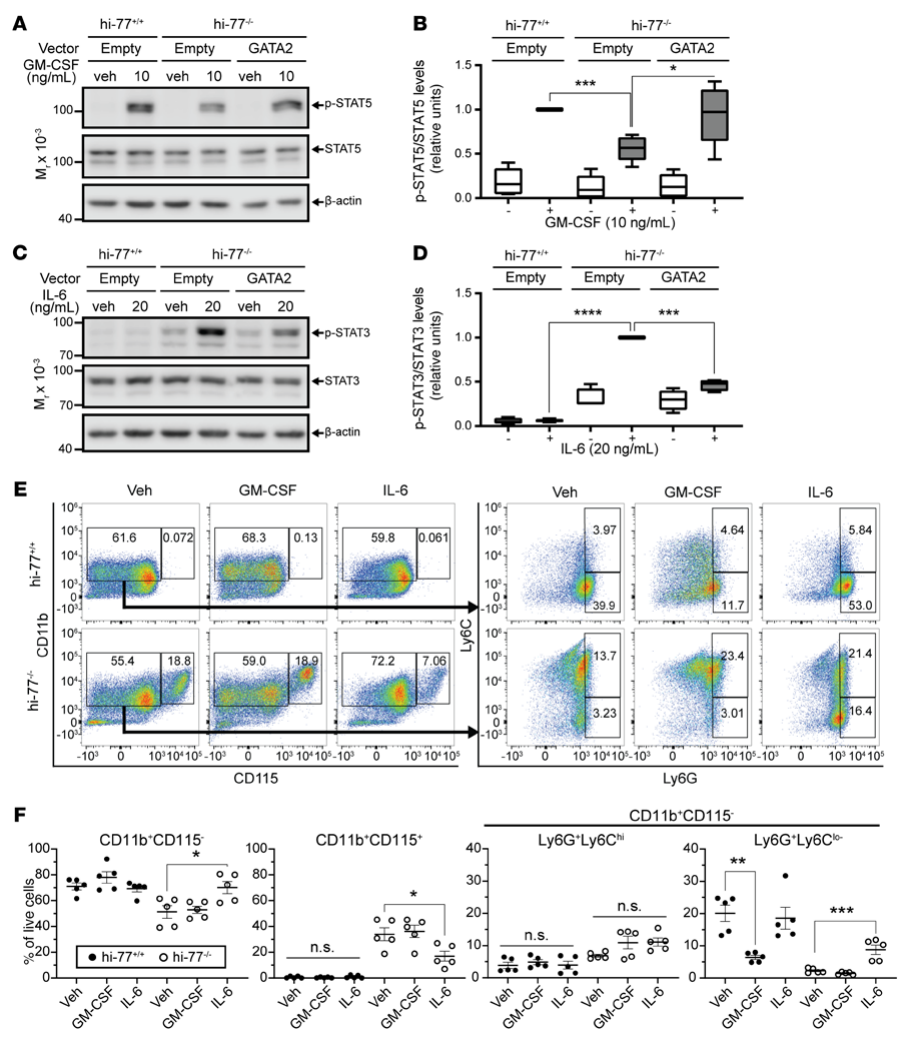
GATA2-mediated regulation of cellular signaling and differentiation. (**A**) Western blot to detect GM-CSF–induced STAT5 phosphorylation (*n* = 6). (**B**) p-STAT5 quantification. Results were normalized to GM-CSF–treated hi–77^+/+^ empty (box-and-whisker plots with bounds from the 25th to the 75th percentiles, the median line, and whiskers ranging from minimum to maximum values) (*n* = 6). (**C**) Western blot to detect IL-6–induced STAT3 phosphorylation (p-STAT3) (*n* = 4). (**D**) p-STAT3 quantification. Results were normalized to IL-6–treated hi–77^–/–^ empty (box-and-whisker plots with bounds from the 25th to the 75th percentiles, the median line, and whiskers ranging from minimum to maximum values) (*n* = 4). Statistical comparisons in **B** and **D** used paired 2-tailed Student’s *t* tests with Benjamini-Hochberg correction; *P < 0.05; ***P < 0.001; ****P < 0.0001. (**E**) Flow cytometric plots of CD11b^+^CD115^–^ (granulocytic) and CD11b^+^CD115^+^ (monocytic) differentiated progenitors cultured for 3 days in control, GM-CSF–containing, or IL-6–containing media (*n* = 5). Plots (gated at CD11b^+^CD115^–^) of Ly6G^+^Ly6C^hi^ and Ly6G^+^Ly6^lo–^ differentiated progenitors cultured for 3 days in control, GM-CSF–containing, or IL-6–containing media (*n* = 5). (**F**) Quantification of CD11b^+^CD115^–^, CD11b^+^CD115^+^, CD11b^+^CD115^–^Ly6G^+^Ly6G^hi^, and CD11b^+^CD115^–^Ly6G^+^Ly6^lo–^ populations. Error bars represent mean ± SEM. For multiple comparisons with vehicle-treated control, statistics were calculated using 1-way ANOVA followed by Dunnett’s test; *P < 0.05; **P < 0.01; ***P < 0.001.

We analyzed GM-CSF signaling with hi–77^+/+^ empty, hi–77^–/–^ empty, and hi–77^–/–^ GATA2 cells treated with vehicle (veh) or GM-CSF for 15 minutes and quantified STAT5 phosphorylation (p-STAT5) (Figure 10, A and B). In GM-CSF–treated cells, the p-STAT5/total STAT5 ratio in hi–77^+/+^ empty cells increased versus hi–77^–/–^ empty cells. Signaling was indistinguishable between hi–77^+/+^ empty and hi–77^–/–^ GATA2 cells, demonstrating that GATA2 elevates signaling. As GATA2 increased *Csf2rb* expression and GM-CSF signaling, and GATA2-deficient progenitors had reduced signaling, these results inform the molecular underpinnings of a pathogenic phenotype.

Commensurate with upregulated *Il6ra* and *Il6st* mRNA levels (Figure 9C), IL-6/STAT3 signaling was upregulated. A 15-minute treatment with IL-6 increased STAT3 phosphorylation (p-STAT3) in hi–77^–/–^ empty, but not hi–77^–/–^ GATA2, cells (Figure 10, C and D). IL-6 induced p-STAT3 in hi–77^–/–^ 9aa-Ins, but not hi–77^–/–^ GATA2, cells (Supplemental Figure 8, A and B). As elevated IL-6 signaling promotes bone marrow failure and MDS to AML progression (50), high IL-6 signaling in GATA2-deficient fetal progenitors may have pathogenic implications.

We tested whether defective GM-CSF and IL-6 signaling impacts differentiation. hi–77^+/+^ versus hi–77^–/–^ cells were treated with GM-CSF or IL-6, and granulocytic and monocytic differentiation was analyzed by flow cytometry with CD11b, CD115, Ly6C, and Ly6G markers (Supplemental Figure 9, A and B). Cells were cultured for 3 days with GM-CSF or IL-6. WT and hi–77^–/–^ cells exhibited greater granulocytic (CD11b^+^CD115^–^) and monocytic (CD11b^+^CD115^+^) differentiation, respectively (Figure 10, E and F). In WT cells, GM-CSF reduced CD11b^+^CD115^–^Ly6G^+^Ly6C^lo–^ granulocytic cells (Figure 10, E and F). GM-CSF treatment of hi–77^–/–^ cells had no effect. IL-6 suppressed the generation of CD11b^+^CD115^+^ monocytic cells to a greater extent with hi–77^–/–^ (Figure 10, E and F) versus WT cells and increased CD11b^+^CD115^–^Ly6G^+^Ly6C^lo–^ granulocytic cells (Figure 10, E and F). Thus, GATA2 deficiency in fetal progenitors generated an aberrant transcriptome that dysregulated progenitor responsiveness to cytokines.

## Discussion

Coding and noncoding variants elicit loss-of-function, gain-of-function, or composite phenotypes that initiate or promote pathogenesis or create a disease predisposition. Our analysis of how a blood disease–causing variant affects GATA factor activity indicates that GATA2 zinc finger dislocation conforms to a model involving hypomorphic and neomorphic attributes. 9aa-Ins was inactive or had reduced activity at most targets, yet it retained activity at a minority of targets and acquired ectopic activity. 8aa-Ins was defective, 2aa-Ins functioned normally, and 4 and 6aa-Ins preferentially disrupted activation.

GATA2 activated and repressed a similar number of genes in progenitors, and 9aa-Ins was incapable of remodeling chromatin and regulating transcription at most loci. 9aa-Ins retained activity at a small cohort of GATA2 targets and ectopic loci. An analysis of several target genes for GATA2 disease mutants T354M and R307W suggested a loss-of-function and gain-of-function phenotype (33). Given GATA2 deficiency syndrome variable penetrance and complex phenotypes (44, 45), hypomorphic and neomorphic attributes may contribute to this complexity. Frameshift mutations can ablate one allele (30), and epigenetic repression of the second allele can exacerbate phenotypes (85). Considering the approximately 2,000 GATA2-regulated genes, lowering GATA2, ectopic activity, or both may corrupt GATA2 genetic networks.

As GATA2-mediated repression is not understood, our demonstration that regulated chromatin sites at GATA2-activated versus -repressed genes were differentially enriched in WGATAR motifs was surprising. Sites at activated genes harbored more WGATAR than sites at repressed genes. GATA1 and GATA2 colocalize on chromatin with other transcription factors and coregulators at sites that often, but not always, contain E-box–spacer–WGATAR composite elements (16, 61, 86–90). The ETS factor FLI1 occupies these sites and is implicated in GATA1- and GATA2-mediated activation (91, 92). Although the ETS factor PU.1 can antagonize GATA1 or GATA2 (74, 93, 94), it can cooperate with GATA2 in activation (95). We tested whether GATA2 regulates expression of ETS factors in this system, and it did not. EBF motifs were enriched at GATA2-repressed loci, and GATA2 occupied *Ebf1* and decreased expression of EBF1, which promotes B-lineage differentiation (38, 72), and EBF1 target genes, including the B-lineage developmental regulator *Pax5* (96) and V-D-J recombination regulators *Rag1* and *Rag2* (97). Ectopic EBF1 expression sufficed to induce the B-lineage expression program (Figure 7B). Although GATA2 deficiency did not alter PU.1 levels, lowering PU.1 levels by ablating a PU.1 enhancer, in the context of *Gata2* –77 enhancer loss, attenuated B-lineage gene upregulation (Figure 7D). PU.1 occupied select genes that were upregulated in GATA2-deficient progenitors. Besides GATA2 inducing hematopoietic stem cell (HSC) generation during embryogenesis, maintaining HSCs in adults and promoting myeloerythroid progenitor differentiation (11), our results support a paradigm in which GATA2 suppresses a B-lineage program that may oppose myeloerythroid differentiation. In B cells, Bach1 and Bach2 suppress a myeloid program (98). As not all GATA2-repressed genes exhibit B lineage–enriched expression, this mechanism constitutes one mode of GATA2-mediated repression, but other mechanisms likely operate at distinct target genes.

GATA2 controls hematopoiesis through cell-intrinsic activities in hematopoietic stem and progenitor cells (HSPCs) (99, 100), yet many questions remain regarding the physiological and pathological mechanisms. *Gata2* mutant transcriptomes (21, 29, 55–57, 83, 101–103), *GATA2* mutant patient samples (104), and GATA2 chromatin occupancy (16, 105, 106) have revealed GATA2-activated and -repressed genes. Despite inferences from gene expression alterations, critical mediators of GATA2 activities are unknown. In GATA2-deficient fetal progenitors, with or without 9aa-Ins, inflammatory and innate immune components were upregulated. In a double-knockout rescue system to test whether upregulation of the innate immune gene activator IRF8 (107) underlies defective differentiation of –77^–/–^ progenitors (108), ablating *Irf8* partially but inefficiently rescued granulopoiesis without restoring erythropoiesis (108). Thus, a GATA2-dependent, IRF8-independent mechanism is also important. The dysregulated cytokine receptors described herein (Supplemental Table 4) may alter GATA2-deficient progenitor responsiveness to extrinsic signals, and our analyses detected such defects.

GATA2, but not 9aa-Ins, normalized cytokine receptor gene expression in GATA2-deficient fetal progenitors. The upregulated genes included those encoding IL-6 receptor subunits. By contrast to *Il6ra*, encoding a dedicated IL-6 receptor subunit, *Il6st* encodes IL6ST (GP130), a subunit of multiple IL-6/IL-12 receptor family members (47, 109). Inflammatory stimuli increase IL-6 elaboration from bone marrow microenvironment cells (48). IL-6 acts on HSPCs to divert functions into emergency granulopoiesis, in which granulocyte-monocyte progenitors (GMPs) disproportionately generate neutrophils (110). In GATA2-deficient fetal progenitors, *Il6ra* and *Il6st* upregulation endowed progenitors with IL-6 signaling, which counteracted the monocytic fate, enabling granulopoiesis, commensurate with IL-6 promotion of granulopoiesis in vivo.

Besides granulopoiesis, high IL-6 induces anemia of inflammation (111, 112). IL-1β pro-proliferative actions on AML progenitors induce inflammatory cytokines including IL-6 (113). AML cell–derived IL-6 was linked to bone marrow failure in a xenograft model. Antibody-mediated neutralization of IL-6 reversed anemia, extending survival (84), and IL-6 promotes MDS progression to AML (50). As elevated IL-6 signaling in GATA2-deficient fetal progenitors suppresses hematopoiesis, in principle, IL-6 may mediate GATA2 pathologies during fetal development.

**Figure 11:**
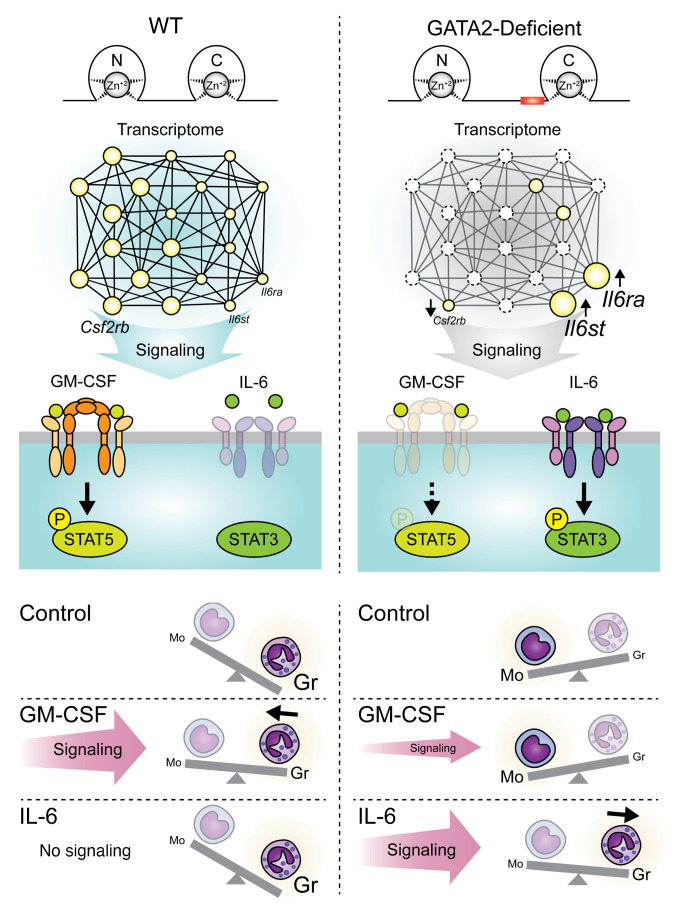
Model of GATA2-regulated genome function, cytokine signaling, and progenitor differentiation. GATA2 deficiency disrupts progenitor cell genome regulation. GATA2 loss decreases *Csf2rb* expression and GM-CSF signaling. GATA2, but not 9aa-Ins, elevates *Csf2rb* expression. GATA2 loss elevates *Il6ra* and *Il6st* expression and IL-6 signaling. WT GATA2, but not 9aa-Ins, reduces *Il6ra* and *Il6st* expression. These alterations impact differentiation and may impact function of progenitor-derived progeny. Normal progenitors exhibit predominantly granulocytic potential, and GM-CSF promotes granulopoiesis. IL-6 does not induce signaling in WT progenitors nor impact differentiation. GATA2-deficient progenitors exhibit predominantly monocytic (Mo) potential, and IL-6 promotes granulocytic (Gr), at the expense of monocytic, differentiation. GM-CSF does not induce signaling in GATA2-deficient progenitors nor impact differentiation. As CSF2RB and IL6ST are shared by additional receptors, their dysregulation will impact a broader ensemble of signaling systems to yield an aberrant network that may disrupt fetal hematopoiesis.

The mechanisms of how GATA2 deficiency and pathogenic GATA2 variants impact processes during human fetal development are not established. Considering proliferative fetal versus quiescent bone marrow HSCs (114, 115) and differential sensitivity of fetal and adult HSCs to FLT3-ITD-induced oncogenesis (116), fetal and adult HSC genomes may differ in sensitivity to dysregulated GATA2. With IL-6, granulopoiesis persisted with GATA2-deficient fetal progenitors (Figure 11), and neutrophils exist in pediatric and adult GATA2 deficiency patients, despite dendritic cell, monocyte, NK cell, and lymphoid cell reductions (44, 45). The neutrophil persistence may reflect sustained granulopoietic activity in GATA2-deficient myeloid progenitors when inflammatory cytokines, e.g., IL-6, drive the process. Otherwise, GATA2-deficient fetal progenitors exhibit a monocytic fate ex vivo, although whether the mutant progeny function normally is unknown (55).

By contrast to elevated IL-6 signaling in GATA2-deficient fetal progenitors, the GM-CSF receptor common β subunit (CSF2RB) decreased, reducing GM-CSF signaling. Whereas GM-CSF induced myeloid differentiation of WT progenitors, signaling-defective GATA2-deficient progenitors were less responsive (Figure 11). CSF2RB is an IL-3 and IL-6 receptor subunit (39–41), and its deficiency would impact signaling networks involving multiple cytokine receptors, analogous to multicomponent aberrations expected from elevated IL6ST, shared by IL-6/IL-12 receptor family members (49). During inflammation, GM-CSF signaling, requiring CSF2RB, elevates neutrophils, monocytes, eosinophils, dendritic cells, and myeloid-derived suppressor cells (117). CSF2RB mediates FLT3-ITD leukemogenic activity by promoting STAT5 phosphorylation (118).

Attenuated GM-CSF signaling and acquired IL-6 signaling, with each system linked to additional cytokine receptors, illustrate complex signaling perturbations caused by GATA2 deficiency in fetal progenitors. 9aa-Ins ectopically repressed expression of cytokine receptors (*Il7ra* and *Il27ra*) and increased *Pglrp1* expression, encoding a conserved peptidoglycan binding protein implicated in *Drosophila* Toll-like receptor (119) and mammalian (120) TNF receptor-1 regulation (Supplemental Table 2). 9aa-Ins increased *Mrgpra2b* expression, encoding an unstudied member of the Itch G protein–coupled receptor family (121) with high expression in myeloid progenitors (haemosphere.org). 9aa-Ins exacerbates the scope of dysregulated signaling in GATA2-deficient fetal progenitors. It is attractive to propose that in GATA2-deficient patients, defective signaling networks attenuate progenitor responsiveness to stress-derived signals, e.g., from pathogens, and responsiveness to other genetic or epigenetic aberrations, thereby causing or contributing to a bone marrow failure and leukemia predisposition.

## Methods

Additional details can be found in Supplemental Methods.

### Immortalized cell culture.

ER-HoxB8-immortalized (hi) progenitors were generated from mouse fetal liver Lin^−^ cells immortalized by retroviral expression of estrogen-regulated HoxB8 (122). Cells were cultured in Opti-MEM (Gibco, Thermo Fisher Scientific) with 10% FBS, 1% penicillin-streptomycin, 1% SCF-conditioned medium, 30 mM β-mercaptoethanol, 1 μM β-estradiol, and 500 μg/mL G418. Cells were cultured in a humidified 5% CO_2_ incubator at 37°C.

### GATA2 rescue assay.

GATA2 or spacer mutants were expressed in –77 enhancer–deleted GATA2-depleted –77^−/−^ hematopoietic progenitors by infection of hi–77^–/–^ cells with retrovirus harboring murine *Gata2* or mutant cDNA in the murine stem cell virus plasmid with GFP (MSCV-PIG; ref. 33). Ecotropic virus was packaged in 293T cells, and retrovirus-containing supernatants were collected 48 hours after transfection. Cells were transferred to IMDM containing 2% FBS and incubated with supernatant by spinoculation for 90 minutes at 1,315*g* at 30°C. Cells were cultured for 3 days in media described above. GFP^+^ and GFP^–^ cells were subjected to protein analysis. GFP^+^ cells were sorted by FACS with a FACSAria cell sorter (BD Biosciences), and RNA was isolated with TRIzol (Invitrogen, Thermo Fisher Scientific).

### Genomic analysis.

Sample preparation and data processing of RNA-Seq (Gene Expression Omnibus [GEO] GSE199464), ATAC-Seq (GEO GSE201968), CUT&RUN (GEO GSE17138), and CUT&Tag (GEO GSE224904) are described in Supplemental Methods.

### Statistics.

qRT-PCR and flow cytometric analysis results were presented as the mean ± SEM. Statistical comparisons for RT-qPCR used unpaired 2-tailed Student’s *t* tests with Benjamini-Hochberg correction. For quantitative flow cytometric analysis, Dunnett’s multiple-comparison test was performed to vehicle-treated control. Western blot signals were presented as box-and-whisker plots with bounds from the 25th to the 75th percentiles, the median line, and whiskers ranging from minimum to maximum values. Dunnett’s test (for GATA2 rescue) or paired 2-tailed Student’s *t* tests with Benjamini-Hochberg correction (for cytokine signaling) were performed to control conditions. All statistics had significance cutoff of *P* less than 0.05 and were calculated using Prism software (GraphPad Software).

### Study approval.

Animal protocols were approved by the University of Wisconsin–Madison IACUC in accordance with the Association for Assessment and Accreditation of Laboratory Animal Care International regulations.

## Author contributions

MMJ and EHB designed the study and wrote the manuscript with input from all authors. MMJ performed experiments and the analyzed data. SS and SK designed analytical strategies and conducted statistical/computational analyses. RAB generated GATA2 CUT&RUN data. PL analyzed RNA-Seq data. GAB and JX conducted ATAC-Seq. TO and MB conducted CUT&Tag. TO and QZ analyzed CUT&Tag data. ZDJ and PWL facilitated generation of ChIP-Seq data. JPM generated the GATA2 molecular model. JKKL, LY, and JPM conducted EMSA. KRK generated cell lines, HA-GATA2, and 9aa-Ins and conducted ChIP-qPCR. KDJ conducted EBF1 and PU.1 experiments. AAS and KDJ facilitated flow analyses. LAG identified the 9aa-Ins patient.

## Supplementary Material

Supplemental data

Supplemental table 1

Supplemental table 2

## Version 1 02/21/2023

In-Press Preview

## Version 2 04/03/2023

Electronic publication
