# Supplementary material for: Pathogenic human variant that dislocates GATA2 zinc fingers disrupts hematopoietic gene expression and signaling networks
Source: J Clin Invest. 2023 Apr 3;133(7):e162685. doi: 10.1172/JCI162685 (PMC10065080; doi:10.1172/JCI162685)
Supplement: Supplemental data [file jci-133-162685-s131.pdf]

Supplementary Methods

GATA2 molecular structure.

A representation of the double GATA1 zinc finger domain (residues 201-310 of human GATA1) bound to DNA was constructed based on the crystal structure of a GATA1:DNA complex (PDB 3VD6, PMID: 26234528). In the original structure, the inter-zinc finger spacer (residues 241-255) was not observed. This sequence was modelled into the structure using the sculpting mode of PYMOL 2.4 (Schroedinger LLC), in a manner that yielded a geometrically reasonable conformation. No attempt was made to model possible interactions between the spacer and DNA, though some contacts with the DNA backbone are likely, given the six basic residues in this 15-residue stretch. The image was created in PYMOL. Structure predictions of the inter-zinc finger spacer were carried out using the Alphafold implementation at: https://colab.research.google.com/github/sokrypton/ColabFold/blob/main/beta/AlphaFold2_advanced.ipynb

Protein analysis for GATA2 rescue.

Infected hi-77$^{+/+}$ cells with empty vector and hi-77$^{-/-}$ cells with empty vector or GATA2 retroviruses were lysed and boiled for 10 min in SDS lysis buffer. Proteins were resolved on 8% SDS-polyacrylamide gels. Proteins were analyzed by semi-quantitative Western blotting with ECL Plus (Thermo Fisher Scientific) and West Femto (Thermo Fisher Scientific). Antibodies used were rabbit polyclonal anti-GATA2 (1, 2), rabbit monoclonal anti-HA-tag (Cell Signaling Technology, 3724) and mouse monoclonal anti-β-actin (Cell Signaling Technology, 3700). Blots were developed by LI-COR Odyssey Imaging System

(LI-COR Biosciences) and quantified by Image Studio Lite (version 5.2) (LI-COR Biosciences)

Immunofluorescence.

Infected hi-77$^{+/+}$ cells with empty vector and hi-77$^{-/-}$ cells with empty or GATA2 were collected on poly-L-lysine coated slides (Electron Microscopy Sciences) and fixed with 3.7% paraformaldehyde in PBS for 10 min at room temperature. Slides were washed with PBS and permeabilized with 0.2% Triton X-100 for 10 min at room temperature. Washed slides were blocked with 3% BSA in PBS with 0.1% Tween 20 for 1 h at room temperature and incubated with anti-HA in rabbit (Cell Signaling Technology, 3724) in 3% BSA at 4˚C overnight. After washing, slides were incubated with Alexa 594 secondary antibody for 1 h at room temperature, washed and mounted using Vectashield mounting medium with DAPI (Vector Laboratories). Images were acquired with a Nikon A1R-S confocal microscope (Nikon).

Gene expression analysis.

Total RNA was purified from 1-5 $\times$ 10^5 cells with TRIzol (Invitrogen) and 2 μg RNA was treated with DNase I (Thermo Fisher Scientific) for 15 min at room temperature. After heat inactivation of Dnase I with EDTA for 10 min at 65°C, RNA was incubated with 250 ng of a 4:1 mixture of oligo(dT) primers and random hexamer at 68°C for 10 min. RNA/primers were incubated with Moloney murine leukemia virus reverse transcription (M-MLV RT) (Thermo Fisher Scientific), 5X first strand buffer (Thermo Fisher Scientific), 10 mM dithiothreitol (Thermo Fisher Scientific), RNAsin (Promega), and 0.5 mM

deoxynucleoside triphosphates (New England Biolabs) at 42°C for 1 h and then heat inactivated at 95°C for 5 min. Quantitative gene expression analyses was performed by real-time qRT-PCR using Power SYBR Green Master Mix (Applied Biosystems) and analyzed on a ViiA 7 Real-Time PCR System (Applied Biosystems). Control reactions without M-MLV RT yielded little to no signal. Relative expression of mRNA was determined from a standard curve of serial dilutions of cDNA samples, and values were normalized to 18S RNA expression. See Table S5 for primers.

RNA-seq for GATA2 rescue.

Four biological replicates of hi−77$^{+/+}$ cells infected with empty vector, hi−77$^{-/-}$ cells infected with empty vector or GATA2 or 9aa-Ins were harvested and sorted for GFP^{+} cells on a FACSAria II instrument (BD Biosciences). RNA was purified using an RNAeasy Micro Kit (Qiagen). Library sequencing through Illumina TruSeq Stranded Total RNA (rRNA reduction) were prepared by the University of Wisconsin-Madison Gene Expression Center and sequenced using an Illumina NovaSeq 6000 sequencer. Global gene expression changes were evaluated by RNA-seq of four biological replicates each of hi−77$^{+/+}$ infected with empty vector, hi−77$^{-/-}$ infected with empty vector, hi−77$^{-/-}$ infected with GATA2-expressing retrovirus, and hi-77$^{-/-}$ infected with 9aa-Ins-expressing retrovirus. Reads were aligned by STAR (version 2.5.2b) (3) to the mouse genome (version mm10) with GENCODE basic gene annotations (version M22). Gene expression levels were quantified by RSEM (version 1.3.0) (4), and differential expression was analyzed by edgeR (version 3.30.3) (5). A differentially expressed gene was required to have at least two-fold changes and an adjusted p-value < 0.05. Gene ontology analysis

was performed by DAVID (version 6.8) (6). Heatmaps of gene expression levels were prepared using ComplexHeatmap (7). Fragments per kilobase of transcript per million (FPKM) mapped reads values were added by 10^{-3} to avoid taking logarithm on zero.

ATAC-seq for GATA2 rescue.

ATAC-seq was performed as described with modifications (8). Briefly, 5 x 10^4 cells were washed twice in PBS and resuspended in 100 μl lysis buffer (10 mM Tris-HCl, 10 mM NaCl, 3 mM $MgCl_2$, 0.1% IGEPAL CA-630, pH 7.4) with freshly added protease inhibitor cocktail (APExBIO). Nuclei were collected by centrifugation at 500 x *g* for 10 min at 4°C. Nuclei were resuspended in 50 μl tagmentation mix (10 mM TAPS, 5 mM $MgCl_2$, pH 8.0 and 1.75 μl Tn5) and incubated at 37°C for 30 min. Tagmentation reactions were terminated by adding 10 μL 0.2% SDS followed by incubation at 55°C for 7 min. Adapter-tagged DNA was purified using MinElute PCR Purification kit (Qiagen) and amplified using KAPA HiFi Hotstart PCR kit (KAPA). ATAC-seq libraries were sequenced on an Illumina NextSeq500 system using the 75-bp high-output sequencing kit. Raw reads were trimmed to remove adapter sequences using Trim Galore! V0.4.1 (https://www.bioinformatics.babraham.ac.uk/projects/trim_galore/) with the "—nextera" parameter and aligned to mouse (mm10) genome assembly using Bowtie2 with default parameters (9). Alignment files were converted to BAM format and duplicate reads were removed using Picard Tools (http://github.com/broadinstitute/picard/). Tags that uniquely mapped to the genome were used for downstream analysis. Output BAM files were converted to BED format using the "bamtobed" command from BEDTools v.2.29.2 (10). Reads were shifted + 4-bp or – 5-bp for positive and negative strand (11), respectively,

using “awk” command. ATAC-seq peaks were identified from shifted-read BED files using MACS v2.0 (12) with the parameter “—nomodel”. Normalized wiggle files were generated from shifted-read BED files using a custom python script. Bigwig files were then generated using wigToBigWig command with the “-clip” parameter (13).

Peak calling for four biological replicates of hi−77$^{-/-}$ infected with empty vector, hi−77$^{-/-}$ infected with GATA2-expressing retrovirus, and hi-77$^{-/-}$ infected with 9aa-Ins-expressing retrovirus was achieved by MACS2 with the parameter –nolambda. Other parameters were set as default. To ensure that peaks were consistent in each condition, we used IDR (Irreproducible Discovery Rate) to compare peaks from pairs of replicates with --idr-threshold 0.05. Peaks were filtered for the width to be narrower than 1 kb. Using these peaks, a master peak list was created by merging all ATAC-seq peaks that are overlapping. Read counts within each master peak region were retrieved from the aligned BAM files by BEDTools. A count matrix was built to summarize the ATAC-seq read count for each biological sample in each master peak region across all experimental conditions. Differential peaks between hi−77$^{-/-}$ GATA2 vs hi−77$^{-/-}$ empty and hi−77$^{-/-}$ 9aa-Ins vs hi−77$^{-/-}$ empty were identified by R package DESeq2 (14). The resulting p-values were adjusted by Benjamini-Hochberg procedures to control the overall False Discovery Rate.

ATAC-seq peak annotation.

R package ChIPseeker (15) was utilized to annotate the genomic features of differentially accessible peaks where the maximum range of promoter to transcription start site (TSS) was set at 3 kb. The differentially accessible peaks were assigned to the nearest genes, based on the distance of the peak region to the TSS, or to genes where the peak is

overlapping, to build associations between ATAC-seq peaks and genes. We only considered protein-coding genes. Peaks at distal intergenics more than 100 kb away from the TSS were removed. We used the annotation in the barplot, violin plot, and the heatmap. For the peaks used in the violin plot, we only filtered out regions lacking peaks at hi−77$^{-/-}$ empty or hi−77$^{-/-}$ GATA2 when analyzing peaks annotated to genes activated/repressed by only GATA2, and filtered out regions lacking peaks at hi−77$^{-/-}$ empty or hi−77$^{-/-}$ 9aa-Ins when analyzing peaks annotated to genes activated/repressed by only 9aa-Ins. For the violin plot restricted to promoters, we extracted ATAC-seq signals at -2 kb to +100 bp of the gene start for hi−77$^{-/-}$ empty, GATA2, and 9aa-Ins. With those regions from the promoters, or the peaks at +/-100 kb, we used the normalize.quantiles function from preprocessCore package (https://github.com/bmbolstad/preprocessCore) to normalize the signal. We calculated the logarithm of the ratio between hi−77$^{-/-}$ GATA2 or hi−77$^{-/-}$ 9aa-Ins and hi−77$^{-/-}$ empty signal. For the peaks used in the barplot, we categorized them as gain, loss, no change based on differential accessibility (Table S6). After obtaining the peak categorization for each gene, we merged peaks in each gene by the following rules: if there was only "gain", we classified the gene as "gain", if there was only "loss", we classified this gene as "loss", and if both "gain" and "loss" were applicable, we classified this gene as "both", and if none were present, then it was either "open to open" or "closed to closed", based on whether peaks existed in this gene.

ATAC-seq motif analyses.

Peaks categorized as gain, loss, no change based on differential accessibility (Table S6) were used as inputs for the motif analysis. We associated peaks with gain and loss to the

nearest RNA-seq DEG with the same direction of regulation (activation and repression). Instead of merging peaks to be associated to one gene, we utilized all the peaks as the input. HOMER software (version 4.11) (16) was used for motif-based sequence analysis and the findMotifGenome.pl function was used to identify known motifs and *de novo* motifs in the different conditions. All parameters in the function were set as default. Motifs in the enrichment analysis (Figure 5D) were chosen such that q-values were smaller than 0.05 and the percentage of motifs in target sequences was larger than 30%. The top 5 motifs from each comparison are chosen in the discriminative analysis (Figure S4C). The heatmaps depicting motif enrichment were generated using the R package pheatmap (https://www.rdocumentation.org/packages/pheatmap/versions/0.2/topics/pheatmap).

GATA2 CUT&RUN.

Primary fetal liver erythroid progenitors were isolated e12.5-e13.5 C57BL/6 mouse embryos. 5-15 livers were pooled for each experimental replicate, mechanically dissociated in staining buffer (PBS, 0.2% BSA, 5 mM glucose) and strained through a 30 µm strainer. Cells were immunostained at 4°C in the presence of rabbit IgG (200 µg/ml, Jackson Laboratories, 015-000-003) to block Fc receptors. To enrich for early erythroid progenitors, cells were first stained with 5 ug/ml biotin-conjugated anti-Ter119 (BDBiosciences, 553672) for 30 min, before magnetic depletion using streptavidin nanobeads (BioLegend, 480016) following the manufacturer's instructions. Cells were then incubated with 0.5 µg/ml APC-conjugated streptavidin (BD Biosciences, 553672), 0.33 µg/ml PE-Cy7-conjugated anti-CD71 (BioLegend, 113811) and a panel of 5 FITC-conjugated lineage antibodies (anti-CD41, anti-CD45R, anti-CD3e, anti-CD11b and anti-

Ly-6G/6C, all at 1 μg/ml; 553848, 553087, 553061, 557396, 553126; BD Biosciences) for 45 min. Cells were then resuspended in FACS running buffer (staining buffer plus 2 mM EDTA). 0.66 ug/ml Hoechst was added immediately prior to sorting in order to distinguish live cells. Cells were sorted into Eppendorf tubes containing 500 μl RPMI supplemented with 10% FCS using a BD FACSAria Fusion machine with a 100 μM nozzle size. CUT&RUN was carried out as described (17). Concanavalin A magnetic beads were activated by washing twice in binding buffer (20 mM HEPES-KOH pH 7.9, 10 mM KCl, 1 mM $CaCl_2$, 1 mM $MnCl_2$). 100,000 FACS-sorted erythroblasts were washed twice in 1.5 ml of wash buffer (20 mM HEPES pH 7.5, 150 mM NaCl, 0.5 mM spermidine and one Roche Complete Protease Inhibitor tablet), resuspended in 200 µl wash buffer and mixed with 10 μl activated bead suspension and rotated for 5–10 min at RT. Tubes were placed in a magnetic rack, wash buffer was replaced with 200 µl of antibody buffer (wash buffer plus 0.02% digitonin, 2 mM EDTA and 100 ng anti-GATA2 affinity-purified rabbit polyclonal anti-GATA2 antibody (1, 2), and the tubes rotated for 2 h at 4°C. Tubes were placed on a magnetic rack, and the cells were washed once with digitonin buffer (wash buffer plus 0.02% digitonin) before resuspension in 200 µl digitonin buffer. pA-MNase enzyme (kind gift from the Henikoff lab) was added to each tube at a final concentration of 700 ng ml^{-1} and the tubes rotated for 1 h at 4°C. Tubes were placed on a magnetic rack, and cells were washed twice with digitonin buffer before resuspending in 150 µl digitonin buffer. The digestion was initiated by the addition of 3 µl 100 mM $CaCl_2$ to each sample and allowed to proceed for 30 min on ice before quenching with 50 µl 4x stop buffer (680 mM NaCl, 40 mM EDTA, 8 mM EGTA, 0.04% digitonin, 0.1 mg ml^{-1} of RNase A, 0.1 mg ml^{-1} glycogen). Tubes were incubated for 10 min at 37°C, centrifuged for 5 min

at 4°C at 16,000 *x* g. The supernatant was moved to fresh tubes, mixed with 1 µl of 20% SDS and 1.5 µl of 20 mg ml^{-1} proteinase K and incubated for 10 min at 70°C. DNA was purified by phenol-chloroform extraction and ethanol precipitation. Libraries were prepared with the Ultra-II DNA library prep kit (NEB) following the manufacturer's protocol with 13 PCR cycles. The mm9 and mm10 builds of the mouse genome were concatenated with the *E. coli* genome and a new Bowtie index was constructed using bowtie-build. Reads were aligned to the mm9+ecoli and mm10+ecoli indices using Bowtie (version 2.4.1) (9). PCR duplicates were removed using Samtools (version 1.10) (18). Reads from a contaminating PCR amplicon mapping to chr11:32270389-32273090 (mm10) were removed. BigWig files were generated from genome coverage using DeepTools (version 3.4.3) (19) and a scaling factor of 1000/[number of reads mapped to *E. coli* genome].

GATA2 and 9aa-Ins CUT&Tag.

CUT&Tag was conducted as described (20). hi-$77^{-/-}$ cells infected with HA-GATA2 or 9aa-Ins were cultured for 3 days and sorted for live, GFP^{+} cells. Collected cells were pooled and lightly permeabilized with 0.1% formaldehyde in room temperature for 2 min and split into 2 replicates per condition (1.25 x 10^5 cells per replicate). Antibodies used were rabbit polyclonal anti-GATA2 (1, 2) and rabbit monoclonal anti-HA-tag (Cell Signaling Technology, 3724).

CUT&Tag peak annotation.

The CUT&Tag data analysis pipeline was implemented as described (21). Raw reads data were aligned to mouse reference genome (mm10) using Bowtie2 (9). Reads that

were duplicated or mapped to the blacklist regions (22) were removed from the analysis. The resulted bam files were sorted and subjected to MACS3 (12) for peak calling. For visualization of the bind profiles, deepTools (19) bamCoverage was used to generate coverage track (bigWig). Peaks called for MACS3 q-value $1e^{-6}$ in the 2 replicates were merged with HOMER (16) mergePeaks. Differential peak analysis was performed in R using the Bioconductor package Diffbind (23, 24). Peaks with an FDR < 0.05 were considered significantly differentially enriched. Diffbind results were used to merge peaks from prior analysis.

Quantitative chromatin immunoprecipitation (ChIP-qPCR).

ChIP analysis was conducted as described (25). hi-77$^{-/-}$ cells infected with HA-GATA2, 9aa-Ins or control vector were cultured for 3 days with 2 μg/ml puromycin. Samples containing 3 x 10^6 cells were crosslinked with 1% formaldehyde for 10 min. Lysates were immunoprecipitated with rabbit anti-HA antibody (Cell Signaling Technology, 3724) using rabbit normal IgG (Cell Signaling Technology, 2729) as a control. DNA was quantified by real-time PCR (Applied Biosystems Viia 7 instrument) with SYBR green fluorescence, and product was quantified relative to a standard curve created from serial dilution of input chromatin.

GATA2 and 9aa-Ins dual zinc finger domain protein production.

GATA2 residues 291-399 and 9aa-Ins residues 291-399 containing the 9aa insertion were cloned into a modified pET15b-based vector. This vector uses the improved translation initiation region TIR-2 (26), expresses proteins without tags but adds 'Met-Gln-

Leu' on the N-terminus as a result of the TIR-2 sequence. Proteins were expressed in *E. coli* BL21(DE3) Rosetta 2 cells for 24 h using autoinduction (27). Cells were lysed by sonication in 50 mM Tris, 100 mM NaCl, 40 μM $ZnSO_4$, 0.5 mM PMSF, 0.1% (v/v) β-mercaptoethanol, pH 7.4, DNase I (10 μg/mL), RNase A (10 μg/mL) and $MgCl_2$ (10 mM). The lysate was clarified by centrifugation at 15,000 × g for 20 min at 4 °C. The soluble fraction was applied to SP Sepharose Fast Flow (Sigma-Aldrich) resin, and protein was eluted with a NaCl step gradient (0.2–1 M). The NaCl concentration in the eluates containing crude GATA2 proteins was adjusted to 0.2 M, and the protein was purified by cation exchange chromatography on a Uno S-1 column (BioRad) in 50 mM Tris, 40 μM $ZnSO_4$, 1 mM DTT, pH 7.4 and eluted using a gradient comprising 0.1–0.8 M NaCl. Proteins were dialyzed into 50 mM Tris, 150 mM NaCl, 1 mM DTT, pH 7.4 and were ~80% pure based on SDS-PAGE analysis. Protein folding was confirmed by analyzing one-dimensional ^{1}H NMR spectra (600 MHz) in 50 mM Tris, 150 mM NaCl, 1 mM DTT, 5% D_2O (v/v), 60 μM DSS pH 7.4.

Electrophoretic mobility shift assay.

Oligonucleotides were obtained at ~90% purity from IDT (Integrated DNA Technologies) and contained a 5' 6-carboxyfluorescein (6-FAM) on one strand. Complementary pairs of oligonucleotides in 1× TE (10 mM Tris, 1 mM EDTA, pH 8) were annealed by heating at 100 °C for 1 min, followed by cooling slowly to room temperature to generate dsDNA. Proteins were added to dsDNA (10 nM or 5 nM) in 1× EMSA buffer [10 mM MOPS, 50 mM KCl, 5 mM $MgCl_2$, 10% (v/v) glycerol, pH 7], incubated on ice for 30 min, and analyzed on 10% (w/v) 19:1 polyacrylamide gels in 0.5× TB buffer (45 mM Tris, 45 mM boric acid, pH 8.3) at 200 V for 45 mins. Gels were imaged on a Typhoon FLA9000 (GE Healthcare)

scanner, using an excitation wavelength of 473 nm and detected using a >510 nm long pass filter.

Ectopic expression of EBF1.

Hemagglutinin (HA)-tagged *Ebf1* coding sequence, corresponding to isoform 2 (NM_007897.3), was amplified from a hi-77 cDNA library using the primers TTTTCTCGAGATGTACCCATACGATGTTCCAGATTACGCTTTTGGGATCCAGGAAAGCATC and AACTATCACATGGGAGGGACAATCATG and cloned into *Xho*I and *Hpa*I sites of the MSCV-PIG retroviral vector. HA-EBF1-expressing and empty retrovirus were packaged in HEK 293T cells and infectious supernatant collected 48 h after transfection. hi-77$^{+/+}$ cells were infected by spinoculation and cultured for 3 d prior to sorting live GFP^{+} cells.

CRISPR-mediated knockout of the Spi1 -14 kb URE

Cas9 guide RNAs flanking the *Spi1* -14b URE were assembled from crRNAs (GTGGCGGACCAAGGACCTCG and CCAAGACTAGGACTCAATAT) annealed to Tracr RNA (Integrated DNA Technologies). The guide RNAs were complexed with Cas9 (Integrated DNA Technologies), and the complex was nucleofected (Amaxa) into hi-77$^{-/-}$ cells. Clones were isolated by limiting dilution and evaluated by PCR for URE ablation.

Protein analysis for cytokine signaling.

hi-77$^{+/+}$ cells infected with empty retrovirus and hi-77$^{-/-}$ cells with empty or GATA2 retroviruses were collected for treatment with GM-CSF or IL-6. For comparison of

signaling in retrovirally-infected hi-77$^{-/-}$ cells, GFP^{+} cells were sorted and expanded 2 d prior to treatment. To induce GM-CSF/STAT5 or IL-6/STAT3 signaling, cells were serum-starved for 2 h and treated with10 ng/ml recombinant mouse GM-CSF (R&D Systems) or 20 ng/ml mouse IL-6 (PeproTech) for 15 min. Samples were washed with PBS and boiled for 10 min in sodium dodecyl sulfate (SDS) lysis buffer (50 mM Tris (pH 6.8), 2% β-mercaptoethanol, 2% SDS, 0.1% bromophenol blue, 10% glycerol) containing phosphatase inhibitor cocktail 1 and 2. Proteins were analyzed by semi-quantitative Western blotting with ECL 2 (Thermo Fisher Scientific) and West Femto (Thermo Fisher Scientific). Antibodies used were rabbit polyclonal anti-pSTAT5 (9351), rabbit monoclonal anti-STAT5 (94205), rabbit monoclonal anti-pSTAT3 (9145), rabbit monoclonal STAT3 (12640) and mouse monoclonal anti-β-actin (3700) from Cell Signaling Technology. Blots were developed by LI-COR Odyssey Imaging System (LI-COR Biosciences) and quantified by Image Studio Lite (version 5.2) (LI-COR Biosciences).

Cell differentiation and flow cytometry.

To induce differentiation, hi-77$^{+/+}$ and hi-77$^{-/-}$ cells were pelleted and washed with PBS and resuspended in differentiation medium consisting of OPTI-MEM supplemented with 10% FBS, 1% penicillin-streptomycin, 1% SCF-conditioned medium, 1% IL-3-conditioned medium, and 30 mM β-mercaptoethanol. Cells were cultured for 3 d at 37˚C and analyzed for monocytic and granulocytic populations. Prior to staining, cells were pelleted and washed with PBS. Cells were stained at 4˚C for 30 min using 1:200 diluted combinations of PE Cy7-Ly6C (BioLegend), FITC-CD11b (BioLegend), PE-CD115 (BioLegend) and APC-Ly6G (eBioscience) in PBS with 10% FBS. After staining, cells were washed once

with PBS with 2% FBS and analyzed on an Attune™ NxT Flow Cytometer (Thermo Fisher Scientific). Differentiated cell populations were analyzed using FlowJo v10.8.0 software (BD Biosciences).

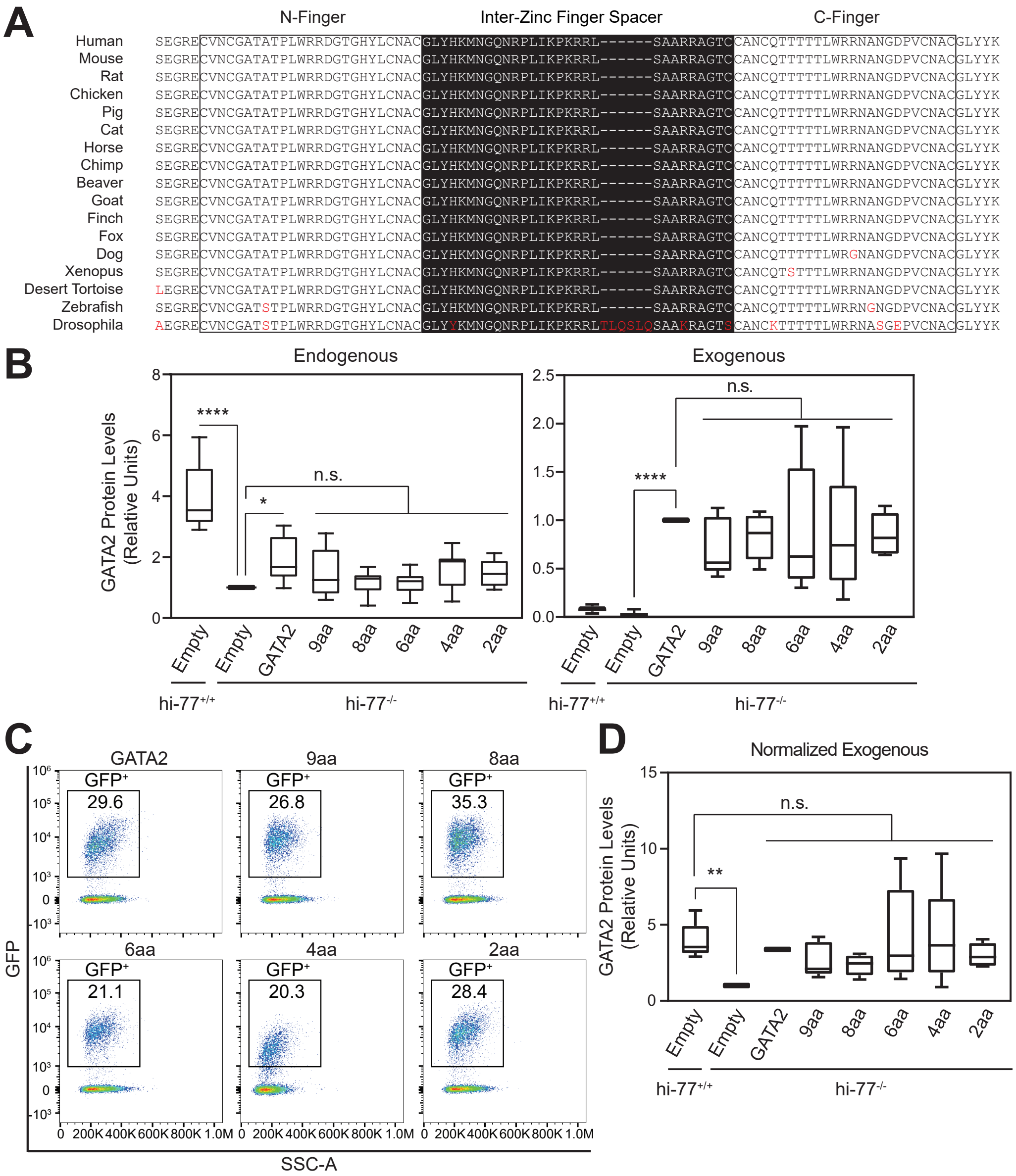

Figure S1. Artificial GATA2 transcription factors with variable inter-zinc finger spacers. (A) Sequence of the GATA2 N-finger, inter-zinc finger spacer, and C-finger among different species. GATA2 homologs Gata2a and Grain were compared for Zebrafish and Drosophila, respectively. All sequences are from UniProt (https://www.uniprot.org/). **(B)** Quantification of endogenous or exogenous GATA2 levels in hi-77 cells with or without expression of GATA2 or variants (n=9). The results were normalized to the hi-77$^{-/-}$ empty condition or the hi-77$^{-/-}$ GATA2 condition. Box-and-whisker plots are plotted with bounds from the 25th to the 75th percentiles, the median line, and whiskers ranging from minimum to maximum values. Endogenous protein levels were compared to hi-77$^{-/-}$ empty. Exogenous protein levels were compared to hi-77$^{-/-}$ GATA2. **(C)** Flow cytometric plots of GFP^{+} hi-77$^{-/-}$ cells expressing HA-tagged GATA2 or variants. **(D)** Quantification of exogenous HA-GATA2 or variants in hi-77$^{-/-}$ normalized based on infection efficiency. Protein levels of endogenous GATA2 in hi-77$^{+/+}$ cells vs. the combination of endogenous and exogenous levels in HA-GATA2 and variants were comparable. Statistical calculations in (B) and (D) utilized one-way ANOVA, followed by Dunnett's test; *, $P < 0.05$; **, $P < 0.01$; ****, $P < 0.0001$; n.s., not significant.

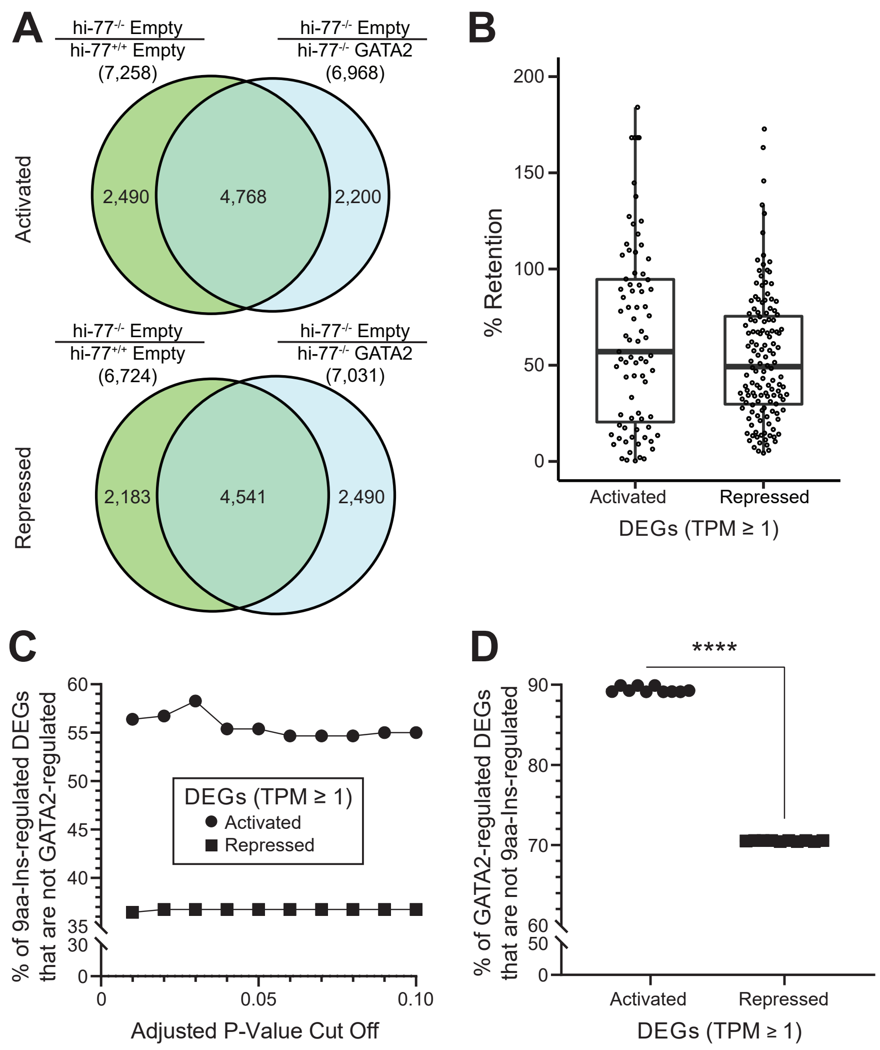

Figure S2. Genome regulation by GATA2 and 9aa-Ins analyzed over a range of statistical stringencies. (A) Overlap of DEGs between (hi-77$^{-/-}$ empty)/(hi-77$^{+/+}$ empty) and (hi-77$^{-/-}$ empty)/(hi-77$^{-/-}$ GATA2) that were $|\log_2(\text{fold change})| > 0$ and no adjusted P-value cutoff deployed. DEGs were parsed into activated and repressed. Genes with fold changes equal to 1 were excluded. **(B)** Percent of 9aa-Ins-regulated genes that are not GATA2-regulated analyzed by subtracting overlap of (hi-77$^{-/-}$ empty)/(hi-77$^{-/-}$ GATA2) from (hi-77$^{-/-}$ empty)/(hi-77$^{-/-}$ 9aa-Ins). Genes were required to have TPM ≥ 1 in all four RNA-seq replicates of at least one of the two conditions under comparison. Different P-cutoffs ranges from 0.01 to 0.1. **(C)** Percent of GATA2-regulated genes not 9aa-Ins-regulated analyzed by subtracting (hi-77$^{-/-}$ empty)/(hi-77$^{-/-}$ 9aa-Ins) from (hi-77$^{-/-}$ empty)/(hi-77$^{-/-}$ GATA2). The same ten P-cutoffs in (A) were used. Genes were required to have TPM ≥ 1 in all of the four RNA-seq replicates of at least one of the two conditions under comparison. Different P-cutoffs ranges from 0.01 to 0.1. The percent of DEGs for each P-cutoff are parsed into activated or repressed. Statistical calculations utilized Mann-Whitney test; ****, $P < 0.0001$. **(D)** Percent retention of 9aa-Ins-regulated genes compared to GATA2. The fold change of (hi-77$^{-/-}$ empty)/(hi-77$^{-/-}$ GATA2) was compared with (hi-77$^{-/-}$ empty)/(hi-77$^{-/-}$ 9aa-Ins). Error bars represent mean ± SEM. Genes were required to have TPM ≥ 1 in four RNA-seq replicates of hi-77$^{-/-}$ GATA2 for activation and hi-77$^{-/-}$ empty for repression.

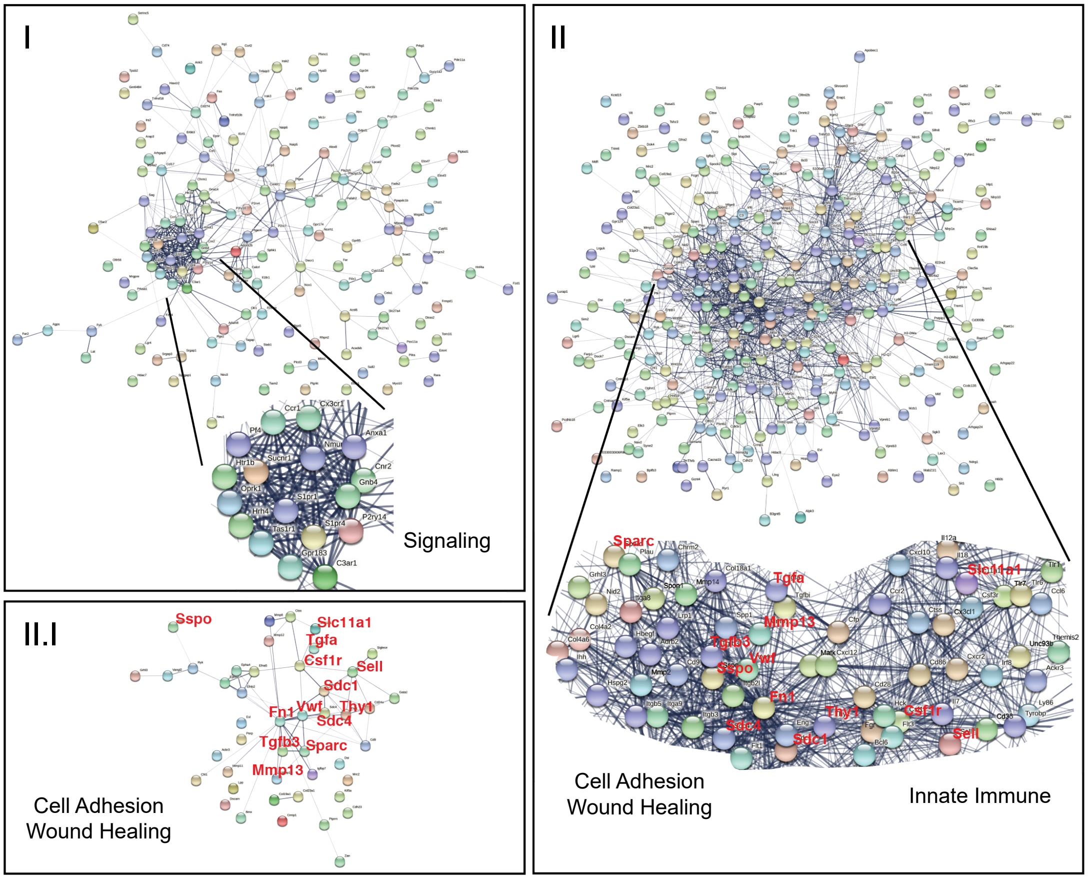

Figure S3. 9aa-Ins mutant is defective in regulating signaling and inflammatory genes. Network Analysis (https://string-db.org/) with genes comprising significant GO terms in each category from Figure 4B.

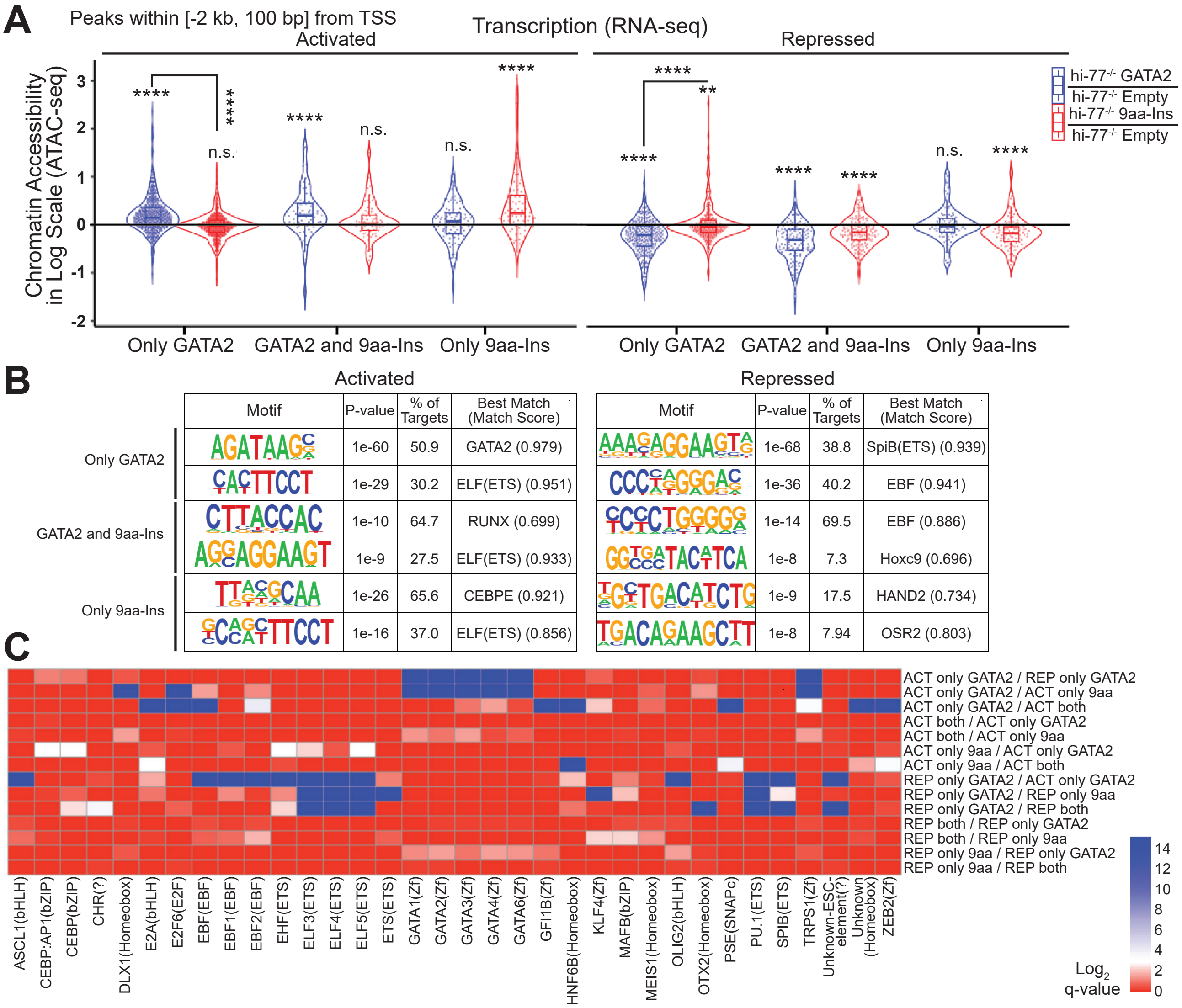

Figure S4. GATA2-regulated chromatin remodeling and transcription. (A) Impact of GATA2 and 9aa-Ins on chromatin accessibility using peaks within -2 kb to +100 bp (promoter) from the start site. hi-77$^{-/-}$ 9aa-Ins/hi-77$^{-/-}$ empty signal, or hi-77$^{-/-}$ GATA2/hi-77$^{-/-}$ empty signal at genes activated or repressed by only GATA2, GATA2 and 9aa-Ins, or only 9aa-Ins by amalgamating all the ATAC-seq peaks associated to RNA-seq genes. Statistical calculations to measure chromatin accessibility (> 0 or < 0 for differential accessibility) utilized Wilcoxon rank sum test. Comparisons between two groups utilized Wilcoxon signed rank test; **, P < 0.01; ****, P < 0.0001; n.s., not significant. **(B)** *De novo* motif enrichment analysis at differentially accessible loci activated or repressed by only GATA2, GATA2 and 9aa-Ins, or only 9aa-Ins. **(C)** Motif discriminative analysis at differentially accessible loci activated or repressed by only GATA2, GATA2 and 9aa-Ins, or only 9aa-Ins. ACT, activated REP, repressed; both, GATA2 and 9aa-Ins.

Gene	hi-77$^{+/+}$ empty	hi-77$^{-/-}$ empty	hi-77$^{-/-}$ GATA2	hi-77$^{-/-}$ 9aa-Ins
Ehf	0.0025	0	0.005	0
Erg	37.45	47.3275	46.58	41.185
Etv1	0	0.005	0	0
Etv2	0.0175	0	0	0
Etv3	6.9075	7.45	6.79	6.2375
Etv3l	0	0	0	0
Etv4	9.3675	2.39	6.9325	2.28
Etv5	5.075	5.77	14.7175	7.165
Etv6	39.1475	44.305	37.2475	43.2325
Elf1	68.8075	95.0175	75.565	73.9825
Elf2	40.2125	46.9575	38.9525	37.3
Elf3	0	0.0025	0	0.0175
Elf4	7.975	9.8375	9.72	8.965
Elf5	0.0075	0	0	0
Elk1	1.955	1.73	2.205	1.5825
Elk3	21.93	28.1425	15.3525	21.8
Elk4	10.4875	12.75	13.4875	10.1475
Fev	0	0	0	0
Spdef	0.0075	0	0.0025	0
Ets1	4.9575	0.84	0.39	0.305
Ets2	6.0225	6.0325	8.185	6.89
Gabpa	37.95	39.415	38.125	37.02
Spi1	26.8075	33.0575	29.6975	37.9525
Spib	0.025	0.05	0.0425	0.0125
Spic	0	0	0	0
Fli1	43.375	41.3725	50.93	46.9075

Table S3. Expression of genes encoding ETS transcription factor family members. Average TPM of 26 genes encoding ETS transcriptions from 4 biological replicates of hi-77$^{+/+}$ empty, hi-77$^{-/-}$ empty, hi-77$^{-/-}$ GATA2, and hi-77$^{-/-}$ 9aa-Ins.

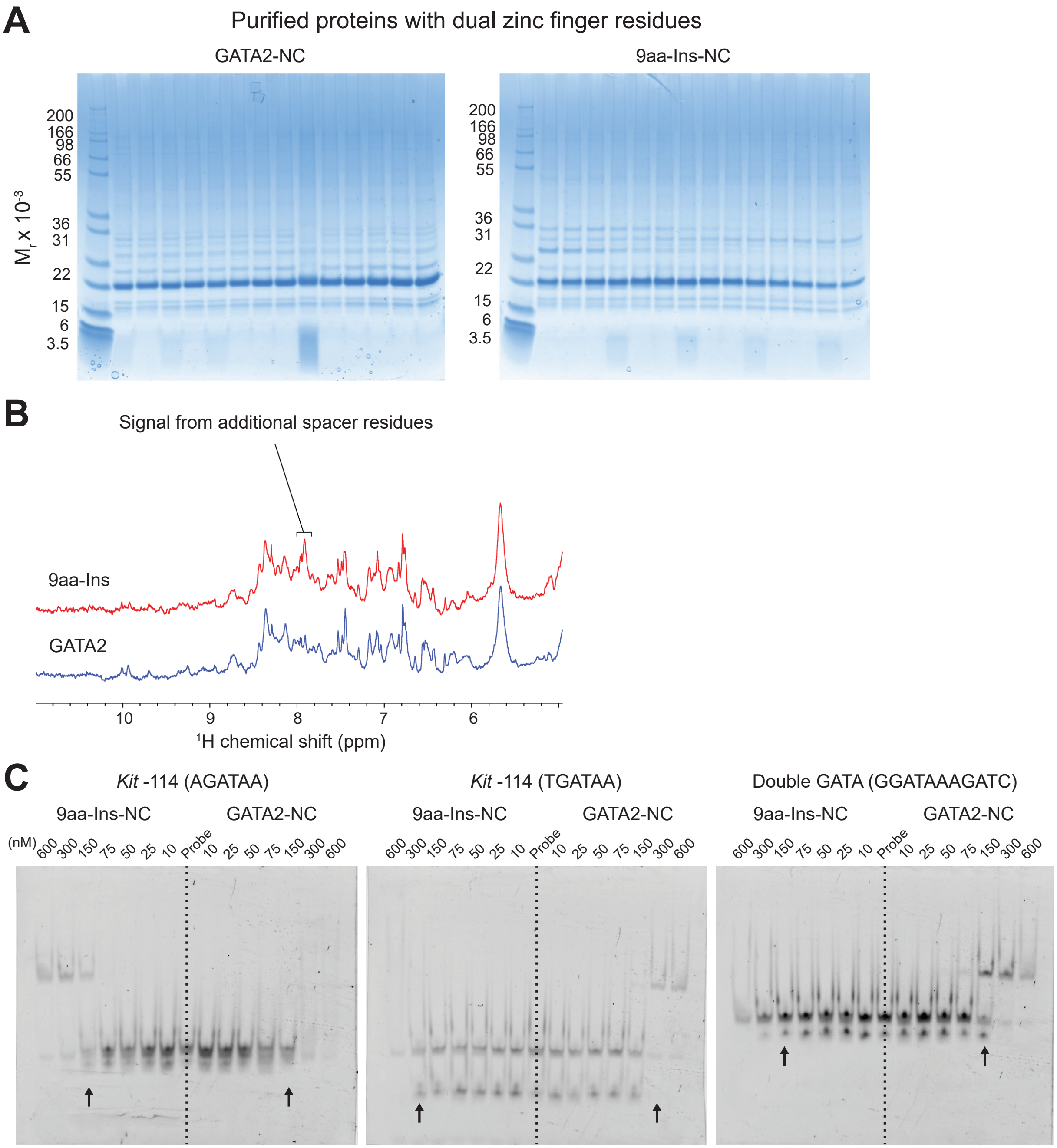

Figure S5. 9aa-Ins mutant has variable influences on naked DNA binding. (A) SDS-PAGE analysis and Coomassie blue staining to assess purity of GATA2 and 9aa-Ins dual zinc finger domain proteins; GATA2 dual zinc finger, GATA2-NC; 9aa-Ins dual zinc finger, 9aa-Ins-NC. The GATA2-NC and 9aa-Ins-NC proteins contain GATA2 291-399 residues or 291-399 residues with 9 insertions between 345 and 346, respectively. **(B)** One-dimensional ^{1}H NMR spectra of amide proton region of GATA2 and 9aa-Ins. **(C)** Electrophoretic mobility shift assay showing probe alone in the center lane and increasing concentrations of each protein in lanes radiating left and right from the center. Arrows show concentration when differences in apparent binding affinities were observed. Compared to WT, 9aa-Ins had a stronger apparent affinity for AGATAA at *Kit* -114kb locus, and an apparent weaker affinity for *Kit* -114 kb TGATAA and double GATA sequence (GGATAAAGATC).

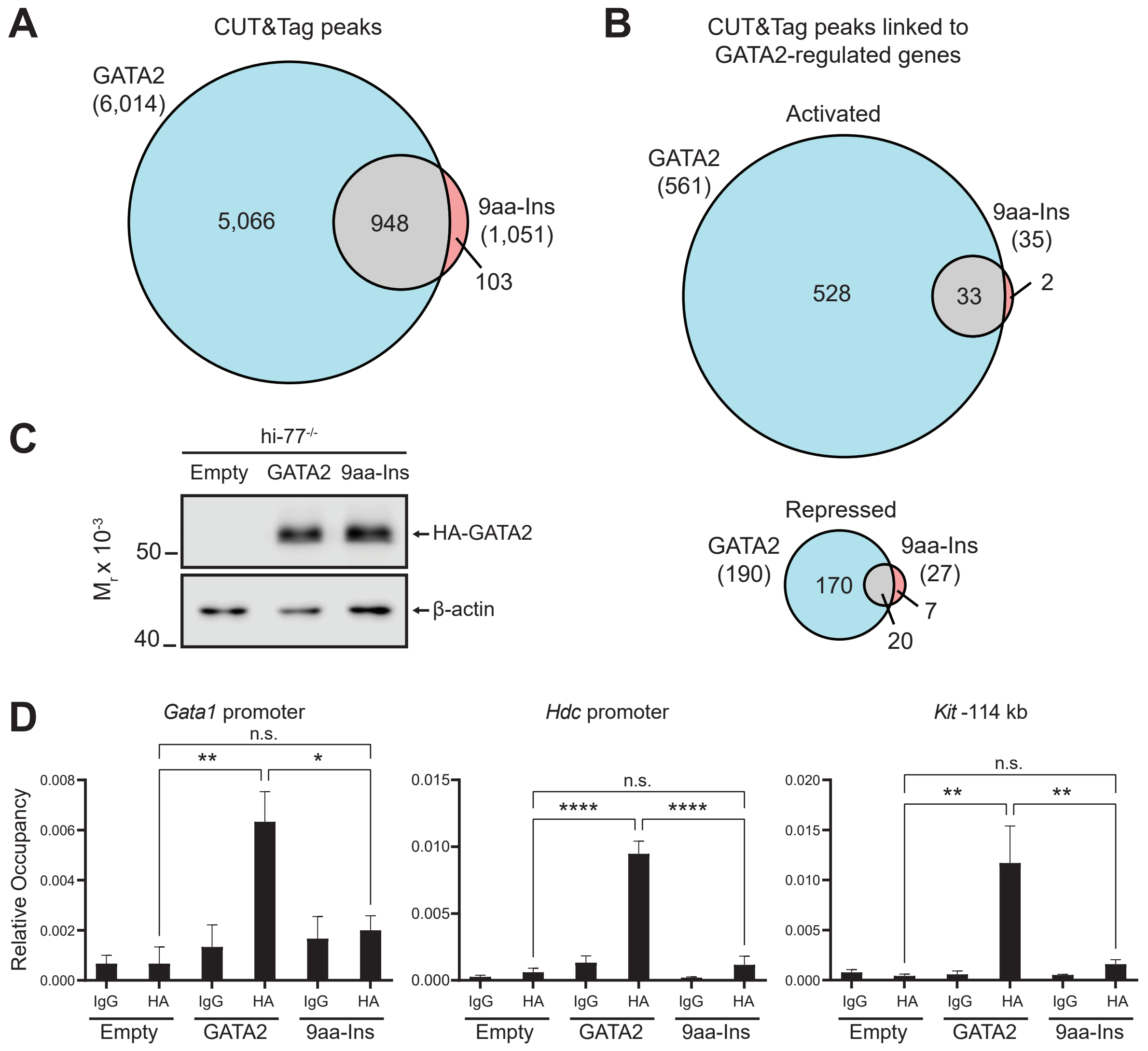

Figure S6. 9aa-Ins mutant substantially loses occupancy in many but not all GATA2 occupancy sites. (A) Venn diagram showing number of CUT&Tag peaks annotated from hi-77$^{-/-}$ GATA2 and hi-77$^{-/-}$ 9aa-Ins. Peaks were called for 2 replicates at MACS3 q-value 1e^{-6} and merged with HOMER mergePeaks and Diffbind. **(B)** Venn diagram showing number of GATA2 and 9aa-Ins CUT&Tag peaks linked to GATA2-regulated genes. Peaks < 100 kb from the gene start site were analyzed. Resulting peaks were parsed into activated and repressed. **(C)** Representative Western blot with anti-HA antibody of hi-77 cells expressing exogenous HA-tagged GATA2 or 9aa-Ins (n=3). **(D)** HA ChIP-qPCR of select three loci (*Gata1* promoter, *Hdc* promoter, *Kit* -114 kb) mined from CUT&Tag analysis (n=3). Statistical calculations utilized unpaired one-way ANOVA, followed by Tukey's test; *, P < 0.05; **, P < 0.01; ****, P < 0.0001

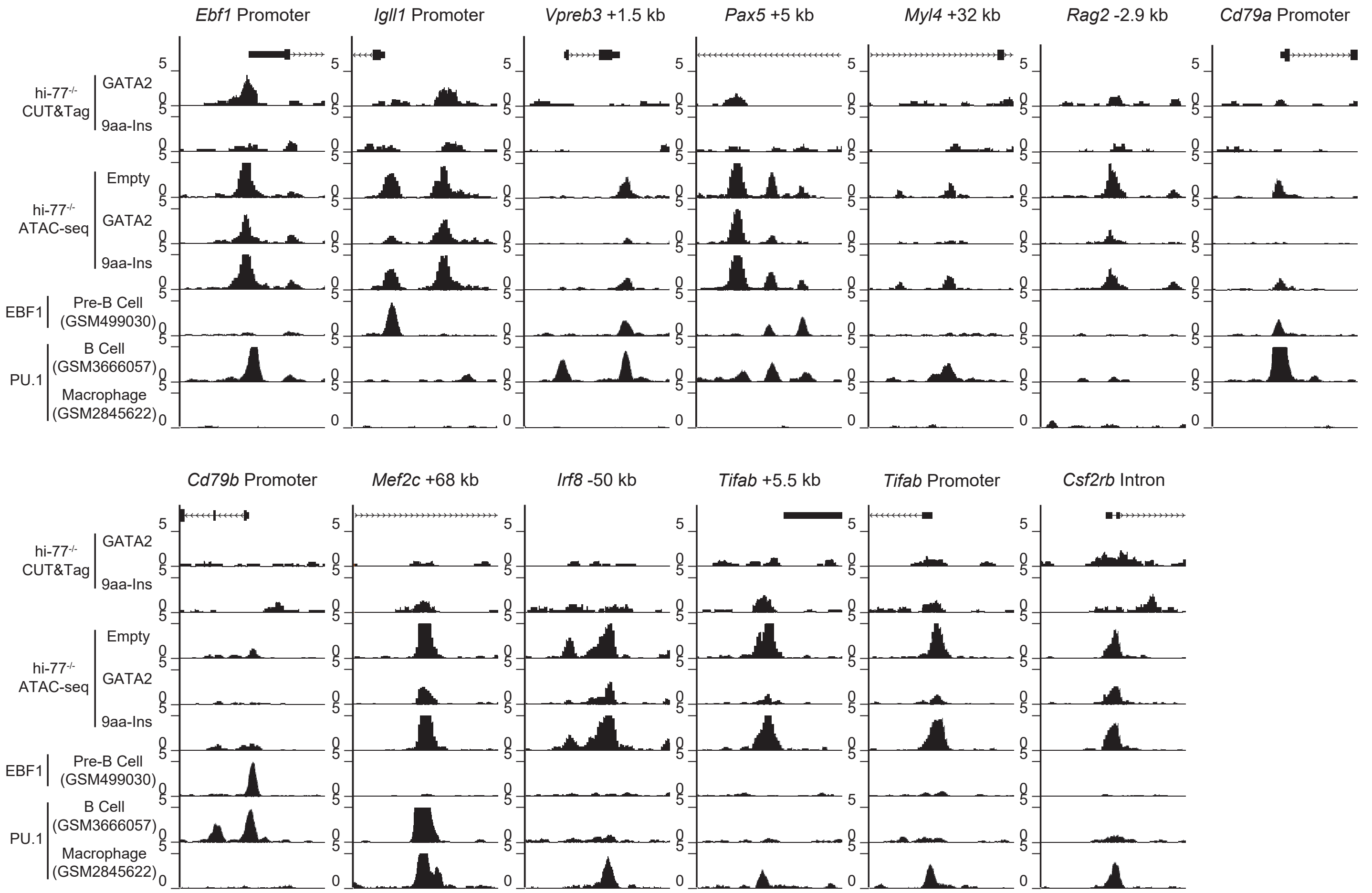

Figure S7. Loci of GATA2-repressed target genes are occupied by EBF and PU.1. **(A)** ChIP-seq profiles for EBF1 and PU.1 in B cell and myeloid lineages mined from existing datasets (GEO: GSM499030, GSM3666057, GSM2845622) compared to the CUT&Tag hi-77$^{-/-}$ GATA2 and hi-77$^{-/-}$ 9aa-Ins profiles and ATAC-seq profiles generated in this study. The -50 kb Irf8 enhancer was previously described (28).

Resource / Gene	ATAC-seq	RNA-seq			
		hi-77	-77 primary	9.5(Ets)	-77 R1
	Chromatin accessibility (│$\log_2$ fold change│)	Gene expression (│$\log_2$ fold change│)			
Il1rl1	n.s.	↑ (+5.7)	↑ (+4.6)	n.s.	↑ (+4.5)
Il3ra	n.s.	n.s.	n.s.	n.s.	↓ (-1.4)
Il5ra	↑ (+2.7)	↑ (+4.5)	↑ (+5.7)	↓ (-5.1)	↓ (-1.1)
Csf2rb	↑ (+1.3)	↑ (+2.9)	↑ (+1.2)	n.s.	↓ (-3.7)
Il6ra	n.s.	↓ (-3.9)	↓ (+1.1)	n.s.	↓ (-4.3)
Il6st	n.s.	↓ (-2.1)	n.s.	n.s.	↓ (-3.4)
Il7r	↓ (-1.1)	n.s.	↑ (+1.2)	↓ (-6.3)	↓ (-3.4)
Il9r	↑ (1.6)	↑ (+5.4)	↑ (+3.6)	↓ (-4.8)	↓ (-1.6)
Il2rg	n.s.	n.s	n.s.	n.s.	↓ (-1.3)
Il10ra	↓ (-2.2)	n.s.	n.s.	n.s.	↓ (-5.4)
Il10rb	↓ (-1.5)	n.s.	n.s.	n.s.	n.s.
Il12rb1	n.s.	n.s.	n.s.	n.s.	↓ (-1.2)
Il13ra1	↓ (-1.7)	n.s.	n.s.	↓ (-4.7)	↓ (-5.4)
Il18r1	n.s.	↑ (+4.5)	↑ (+3.2)	n.s.	n.s.
Il18rap	↓ (-2.3)	↑ (+1.3)	↑ (+1.4)	n.s.	↓ (-1.8)
Il20ra	↓ (-2.2)	n.s.	n.s.	n.s.	n.s.
Il21r	n.s.	n.s.	↓ (-1.1)	n.s.	↓ (-2.5)
Il31ra	↓ (-2.0)	n.s.	↑ (+2.0)	n.s.	↑ (+1.2)

Table S4. Genomic resources that reveal GATA2-dependent multi-cytokine signaling network. ATAC-seq and RNA-seq datasets that revealed GATA2 regulation of interleukin receptors; increased (arrow up) and decreased (arrow down), chromatin accessibility from ATAC-seq, and activation (arrow up) and repression (arrow down) measured by RNA-seq; n.s., not significant. For accessibility, the nearest differentially accessible ATAC-seq peak from the transcriptional start site was chosen.

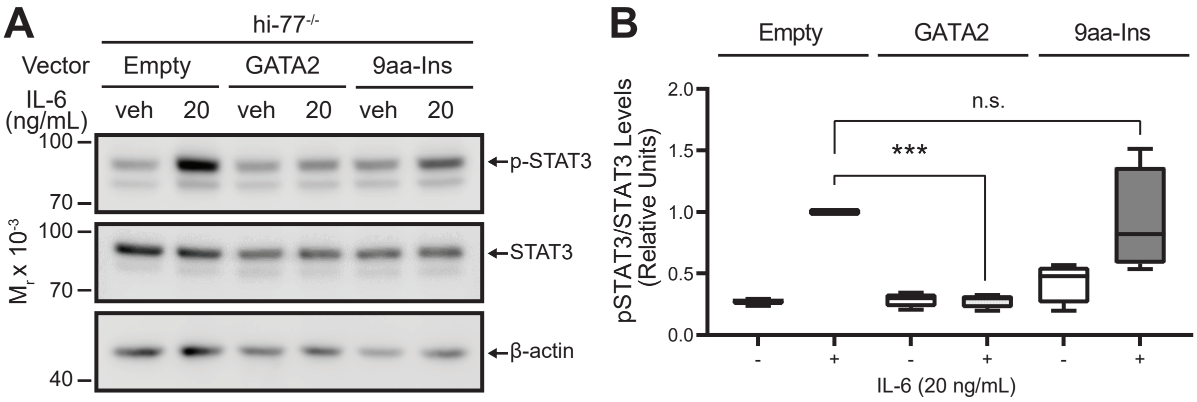

Figure S8. **Dislocating GATA2 zinc fingers impairs GATA2-mediated suppression of IL-6 signaling. (A)** Representative Western blot to detect IL-6-induced STAT3 phosphorylation (p-STAT3) (n = 4) in hi-77$^{-/-}$ GATA2 and hi-77$^{-/-}$ 9aa-Ins compared to hi-77$^{-/-}$ empty control. **(B)** p-STAT3 quantification. The data was normalized to IL-6-treated hi-77$^{-/-}$ empty and presented as box-and whisker plots with bounds from the 25th to the 75th percentiles, the median line, and whiskers ranging from minimum to maximum values (n = 4). Statistical comparisons utilized paired two-tailed Student's t tests with Benjamini-Hochberg correction; ***, $P < 0.001$; n.s., not significant.

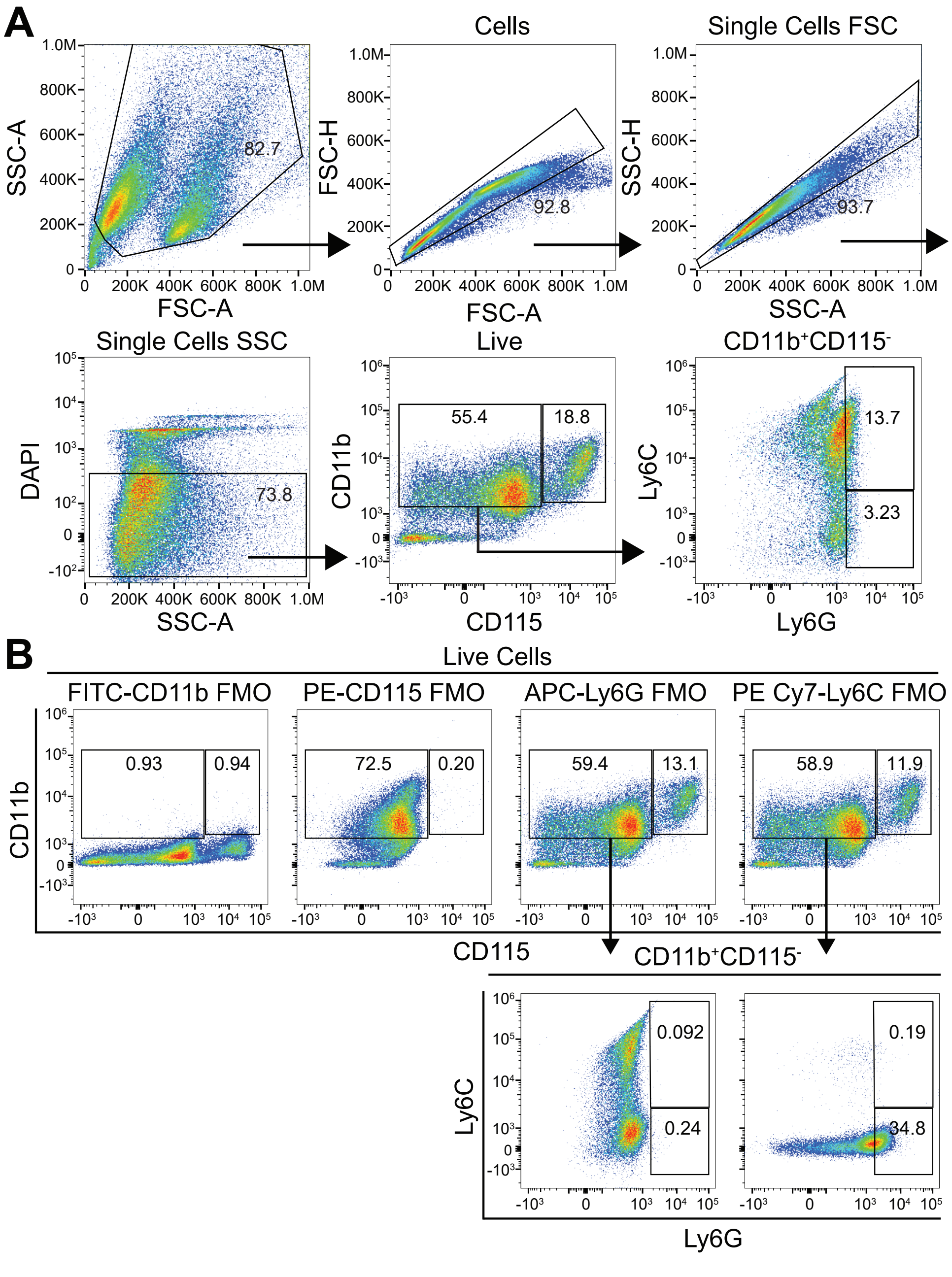

Figure S9. Gating strategy for progenitor differentiation analysis. (A) Flow cytometric gating strategy for hi-77 progenitors to identify populations that undergo granulocytic ($CD11b^{+}CD115^{-}$) or monocytic ($CD11b^{+}CD115^{+}$) differentiation. $CD11b^{+}CD115^{-}$ populations were parsed into $Ly6G^{+}Ly6C^{hi}$ and $Ly6G^{+}Ly6^{lo-}$. The sample shown was vehicle-treated hi-$77^{-/-}$. **(B)** Representative FMOs to guide gating of (A). A combination of vehicle-treated hi-$77^{+/+}$ and hi-$77^{-/-}$ samples was used for FMOs.

Table S5. Primers for qRT-PCR and ChIP-qPCR.

Primers	Species	Sequence (5'->3')
Hdc mRNA	mouse	AGCTGGGACAGCATCTTTGG<br>CAGGATAGTAGGCGTGCATGTG
Gata1 mRNA	mouse	GGCCCAAGAAGCGAATGATT<br>GGTTCACCTGATGGAGCTTGA
Il1rl1 mRNA	mouse	GGCTCTCACTTCTTGGCTGATG<br>CAATCCATGTCATTGCTGGAA
Csf2rb mRNA	mouse	ACATTCCAGGTCCAGTACAAG<br>GTAGTTAGAGATGGGCTTGACC
Irf8 mRNA	mouse	TGCCACTGGTGACCGGATAT<br>GCCCCCGTAGTAGAAGCTGA
Tifab mRNA	mouse	GTCCTGTCTGCTCATCTGTTC<br>GGGCCTCTCCATAGATCCAG
Il6ra mRNA	mouse	CCCTCTCCAACCACGAAG<br>GGTATACTTTGTCACCCTCCAG
Il6st mRNA	mouse	GAAACAAGGTGGGCAAATCAG<br>GGGTTTAGGTGGAGGTGTC
Gata1 promoter ChIP	mouse	CCAGGACTAGTGGGTAAGGGT<br>AGCCCCAAGACAGCCTGTTA
Hdc promoter ChIP	mouse	TGGATGGACGCTTCTACAGC<br>AGTGGCAATTCTTCCCCCTT
Kit -114 kb ChIP	mouse	GCACACAGGACCTGACTCCA<br>GTTCTGAGATGCGGTTGCTG
Ebf1 mRNA	mouse	ATCTGGTTGAAGCCCTGTATG<br>TGGACCGAAGTGTTAGCAAG
Cd79a mRNA	mouse	AAGGTACCAAGAACCGCATC<br>ATGTCCACCCCAAACTTCTC
Cd79b mRNA	mouse	CCCATCTTCCTGCTACTTGAC<br>TCCTACCGACCACTTTACCTC
Myl4 mRNA	mouse	AACACATCTCCCGCAACA<br>CTCTGCCTCGCTCATCTTC
Gata2 mRNA	mouse	GCAGAGAAGCAAGGCTCGC<br>CAGTTGACACACTCCCGGC
Pax5 mRNA	mouse	CACAGTCCTACCCTATTGTCAC<br>TCCAGAAAATTCACTCCCAGG
Igll1 mRNA	mouse	GTTTTGGTATGTCTTTGGTGGTG<br>CCTGGGTAGAATTCGCTCAC
Mef2c mRNA	mouse	CCAGATCTCCGCGTTCTTATC<br>CCTCCCATTCCTTGTCCTG
Vpreb3 mRNA	mouse	TGATAGGAACCTTTGTGGCAG<br>GTACCACGACACCCCAATATC
Rag2 mRNA	mouse	GAGTTTAATTCCTGGCTTGGC<br>GTGAGAAGCCTGGTTGAATTAAG
Spi1 mRNA	mouse	GGCAGCGATGGAGAAAGC<br>GGACATGGTGTGCGGAGAA

Table S6. Categorization of differentially accessible peaks.

Peaks	hi−77$^{-/-}$ GATA2 / hi−77$^{-/-}$ empty		hi−77$^{-/-}$ 9aa-Ins / hi−77$^{-/-}$ empty		Categorization
	Log_2 fold change	P-value	Log_2 fold change	P-value	
GATA2 and 9aa-Ins	< 0	< 0.05	< 0	< 0.05	Loss
	> 0	< 0.05	> 0	< 0.05	Gain
	/	> 0.05	/	> 0.05	No change
Only GATA2	< 0	< 0.05	/	/	Loss
	> 0	< 0.05	/	/	Gain
	/	> 0.05	/	/	No change
Only 9aa-Ins	/	/	< 0	< 0.05	Loss
	/	/	> 0	< 0.05	Gain
	/	/	/	> 0.05	No change

References

1. Im H, Grass JA, Johnson KD, Kim S-I, Boyer ME, Imbalzano AN, et al. Chromatin domain activation via GATA-1 utilization of a small subset of dispersed GATA motifs within a broad chromosomal region. *Proc Natl Acad Sci U S A.* 2005;102:17065-70.
2. Fujiwara T, O'Geen H, Keles S, Blahnik K, Linnemann AK, Kang YA, et al. Discovering hematopoietic mechanisms through genome-wide analysis of GATA factor chromatin occupancy. *Mol Cell.* 2009;36(4):667-81.
3. Dobin A, Davis CA, Schlesinger F, Drenkow J, Zaleski C, Jha S, et al. STAR: ultrafast universal RNA-seq aligner. *Bioinformatics.* 2013;29(1):15-21.
4. Li B, and Dewey CN. RSEM: accurate transcript quantification from RNA-Seq data with or without a reference genome. *BMC Bioinformatics.* 2011;12:323.
5. Robinson MD, McCarthy DJ, and Smyth GK. edgeR: a Bioconductor package for differential expression analysis of digital gene expression data. *Bioinformatics.* 2010;26(1):139-40.
6. Huang DW, Sherman BT, Tan Q, Kir J, Liu D, Bryant D, et al. DAVID Bioinformatics Resources: expanded annotation database and novel algorithms to better extract biology from large gene lists. *Nucleic Acids Res.* 2007;35(Web Server issue):W169-75.
7. Gu Z, Eils R, and Schlesner M. Complex heatmaps reveal patterns and correlations in multidimensional genomic data. *Bioinformatics.* 2016;32(18):2847-9.
8. Liu X, Zhang Y, Chen Y, Li M, Zhou F, Li K, et al. In Situ Capture of Chromatin Interactions by Biotinylated dCas9. *Cell.* 2017;170(5):1028-43 e19.
9. Langmead B, and Salzberg SL. Fast gapped-read alignment with Bowtie 2. *Nat Methods.* 2012;9(4):357-9.
10. Quinlan AR, and Hall IM. BEDTools: a flexible suite of utilities for comparing genomic features. *Bioinformatics.* 2010;26(6):841-2.
11. Yan F, Powell DR, Curtis DJ, and Wong NC. From reads to insight: a hitchhiker's guide to ATAC-seq data analysis. *Genome Biol.* 2020;21(1):22.
12. Zhang Y, Liu T, Meyer CA, Eeckhoute J, Johnson DS, Bernstein BE, et al. Model-based analysis of ChIP-Seq (MACS). *Genome Biol.* 2008;9(9):R137.
13. Kent WJ, Zweig AS, Barber G, Hinrichs AS, and Karolchik D. BigWig and BigBed: enabling browsing of large distributed datasets. *Bioinformatics.* 2010;26(17):2204-7.
14. Love MI, Huber W, and Anders S. Moderated estimation of fold change and dispersion for RNA-seq data with DESeq2. *Genome Biol.* 2014;15(12):550.
15. Yu G, Wang LG, and He QY. ChIPseeker: an R/Bioconductor package for ChIP peak annotation, comparison and visualization. *Bioinformatics.* 2015;31(14):2382-3.
16. Heinz S, Benner C, Spann N, Bertolino E, Lin YC, Laslo P, et al. Simple combinations of lineage-determining transcription factors prime cis-regulatory elements required for macrophage and B cell identities. *Mol Cell.* 2010;38(4):576-89.
17. Skene PJ, and Henikoff S. An efficient targeted nuclease strategy for high-resolution mapping of DNA binding sites. *Elife.* 2017;6.
18. Danecek P, Bonfield JK, Liddle J, Marshall J, Ohan V, Pollard MO, et al. Twelve years of SAMtools and BCFtools. *Gigascience.* 2021;10(2).
19. Ramírez F, Ryan DP, Grüning B, Bhardwaj V, Kilpert F, Richter AS, et al. deepTools2: a next generation web server for deep-sequencing data analysis. *Nucleic Acids Res.* 2016;44(W1):W160-5.
20. Li Y, Nakka K, Olender T, Gingras-Gelinas P, Wong MM, Robinson DCL, et al. Chromatin and transcription factor profiling in rare stem cell populations using CUT&Tag. *STAR Protoc.* 2021;2(3):100751.

21. Kaya-Okur HS, Wu SJ, Codomo CA, Pledger ES, Bryson TD, Henikoff JG, et al. CUT&Tag for efficient epigenomic profiling of small samples and single cells. *Nat Commun.* 2019;10(1):1930.
22. Amemiya HM, Kundaje A, and Boyle AP. The ENCODE Blacklist: Identification of Problematic Regions of the Genome. *Sci Rep.* 2019;9(1):9354.
23. Gentleman RC, Carey VJ, Bates DM, Bolstad B, Dettling M, Dudoit S, et al. Bioconductor: open software development for computational biology and bioinformatics. *Genome Biol.* 2004;5(10):R80.
24. Ross-Innes CS, Stark R, Teschendorff AE, Holmes KA, Ali HR, Dunning MJ, et al. Differential oestrogen receptor binding is associated with clinical outcome in breast cancer. *Nature.* 2012;481(7381):389-93.
25. Katsumura KR, Yang C, Boyer ME, Li L, and Bresnick EH. Molecular basis of crosstalk between oncogenic Ras and the master regulator of hematopoiesis GATA-2. *EMBO Rep.* 2014;15(9):938-47.
26. Shilling PJ, Mirzadeh K, Cumming AJ, Widesheim M, Köck Z, and Daley DO. Improved designs for pET expression plasmids increase protein production yield in Escherichia coli. *Commun Biol.* 2020;3(1):214.
27. Studier FW. Protein production by auto-induction in high density shaking cultures. *Protein Expr Purif.* 2005;41(1):207-34.
28. Schönheit J, Kuhl C, Gebhardt ML, Klett FF, Riemke P, Scheller M, et al. PU.1 level-directed chromatin structure remodeling at the Irf8 gene drives dendritic cell commitment. *Cell Rep.* 2013;3(5):1617-28.
